# How people wake up is associated with previous night’s sleep together with physical activity and food intake

**DOI:** 10.1038/s41467-022-34503-2

**Published:** 2022-11-19

**Authors:** Raphael Vallat, Sarah E. Berry, Neli Tsereteli, Joan Capdevila, Haya Al Khatib, Ana M. Valdes, Linda M. Delahanty, David A. Drew, Andrew T. Chan, Jonathan Wolf, Paul W. Franks, Tim D. Spector, Matthew P. Walker

**Affiliations:** 1 Center for Human Sleep Science, Department of Psychology, University of California, Berkeley, CA USA; 2 Department of Nutritional Sciences, King’s College London, London, UK; 3 Department of Clinical Sciences, Lund University, Malmö, Sweden; 4 Zoe Ltd, London, UK; 5 School of Medicine, University of Nottingham, Nottingham, UK; 6 Nottingham NIHR Biomedical Research Centre, Nottingham, UK; 7 Diabetes Unit, Massachusetts General Hospital and Harvard Medical School, Boston, MA USA; 8 Clinical & Translational Epidemiology Unit, Massachusetts General Hospital and Harvard Medical School, Boston, MA USA; 9 Division of Gastroenterology, Massachusetts General Hospital and Harvard Medical School, Boston, MA USA; 10 Department of Twin Research and Genetic Epidemiology, King’s College London, London, UK; 11 Department of Nutrition, Harvard Chan School of Public Health, Boston, MA USA

**Keywords:** Circadian rhythms and sleep, Heritable quantitative trait, Human behaviour

## Abstract

How people wake up and regain alertness in the hours after sleep is related to how they are sleeping, eating, and exercising. Here, in a prospective longitudinal study of 833 twins and genetically unrelated adults, we demonstrate that how effectively an individual awakens in the hours following sleep is not associated with their genetics, but instead, four independent factors: sleep quantity/quality the night before, physical activity the day prior, a breakfast rich in carbohydrate, and a lower blood glucose response following breakfast. Furthermore, an individual’s set-point of daily alertness is related to the quality of their sleep, their positive emotional state, and their age. Together, these findings reveal a set of non-genetic (i.e., not fixed) factors associated with daily alertness that are modifiable.

## Abstract (web summary)

In a prospective longitudinal study of 833 adults, we demonstrate that how you wake up and regain alertness in the hours after sleep is weakly associated with your genes. Instead, the modifiable factors of how you are sleeping, eating and exercising influence your return to full alertness, free of sleepiness.

## Introduction

What factors will influence how you wake up tomorrow morning, predicting whether or not you will feel alert, and then be able to sustain that level of attentive waking consciousness across the day? This question is scientifically elemental but also of societal relevance considering that the failure to sustain alertness throughout the day is a major causal factor of road traffic and occupational accidents, accounting for thousands of deaths every year^1,2^. Moreover, it is estimated that insufficient sleep leading to impaired daytime alertness is responsible for significant work-related loss of productivity, greater healthcare utilisation and work absenteeism, thereby costing developed nations about 2% of their gross domestic product each year (i.e., $411 billion dollars in the United States alone)^3^.

In addition, the inability to transition effectively to a state of functional cognitive alertness upon awakening from sleep—known as “sleep inertia” ^4^—is a serious safety risk for workers performing hazardous tasks immediately upon awakening, one that has cost such individuals either their own lives or the lives of others (e.g. military personnel, healthcare workers, firefighters, pilots)^5–7^.

Despite the severity, magnitude, and scope of these consequences, the unique factors that influence how each of us wakes up and sustain meaningful alertness throughout a waking day are poorly understood at both the extrinsic and intrinsic (biological) level.

Addressing these issues, here, we sought to test whether a set of a priori factors are associated with alertness in the first hours after awakening from sleep.

Building on previous research, and using the Personalized Responses to Dietary Composition Trial 1 (“PREDICT1”)^8,9^, we targeted four interrelated hypotheses, the motivational evidence for which we describe below. First, we tested the hypothesis that an individual’s unique sleep profile the night prior — i.e., their sleep duration, sleep efficiency, and sleep timing — predicts subsequent changes in next-morning alertness. Specifically, a higher-than-typical sleep duration and sleep efficiency for any one individual would uniquely predict superior (higher) next-day alertness for that individual. Second, higher levels of physical activity on the day prior would predict an increase in next-day morning alertness. Third, that the macronutrient composition of breakfast, and independent of that composition, the unique associated blood glucose response, each selectively influences morning alertness. Fourth, and beyond these modifiable lifestyle factors (i.e., sleep, food, physical activity), we additionally sought to test whether daytime alertness is under significant genetic heritability using twin-pair genetic modeling.

## Results

### Modifiable lifestyle factors are associated with day-to-day fluctuations in morning alertness

**Fig. 1:**
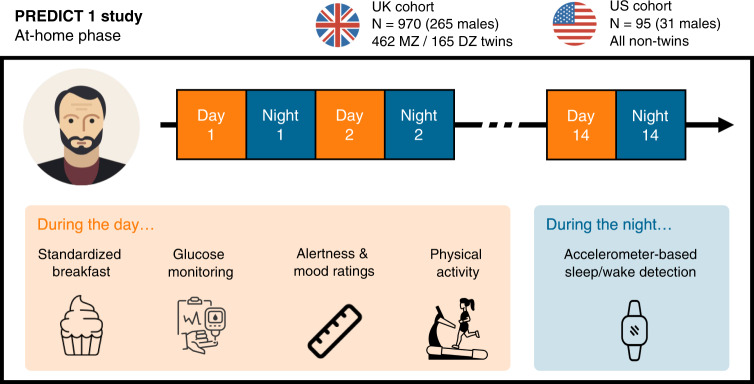
Experimental design. The Personalized Responses to Dietary Composition Trial (or “PREDICT1”) is a two-country (UK, US) longitudinal study whose primary goal is to predict metabolic responses to foods based on the individual’s characteristics, including molecular biomarkers and lifestyle factors, as well as the nutritional composition of the food^8^. PREDICT1 consists of one clinic baseline visit followed by a two-week home-based study. During the at-home phase, participants consumed multiple standardised test meals differing in macronutrient composition, while wearing an accelerometer wristwatch and a continuous glucose monitor. The former was used to determine sleep/wake activity during the night and physical activity during the day. The continuous glucose monitor was used to measure postprandial glucose response. Participants also recorded their dietary intake, satiety, mood, and exercise on the study app throughout the study. The app also prompted participants to report their alertness levels on a 0–100 visual analogue scale at t = 0 minutes (time of logging of a meal) and regular intervals following the logging of a meal (see “Methods”). Source data are provided as a Source Data file. The cupcake icon, the ruler icon and the smartwatch icon were purchased and downloaded from thenounproject.com. All other icons were purchased and/or downloaded from iconfinder.com. MZ monozygotic, DZ dizygotic.

**Fig. 2:**
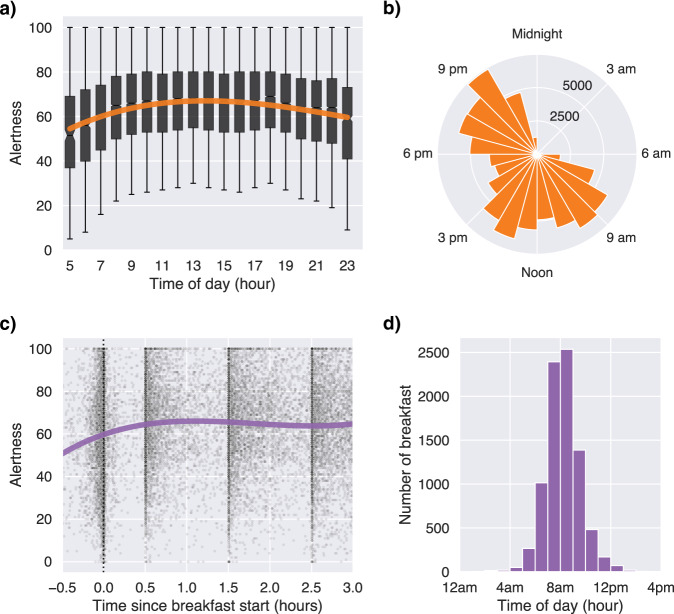
Alertness ratings throughout the day. **a** Alertness as a function of time of day. The orange line shows a cubic regression of all the alertness ratings logged between 5am and midnight (n = 89,440). Alertness progressively increased in the first hours of the morning, reached a plateau during midday and progressively decreased in the evening. Sample size for each unique box is shown in panel B. Box plots show centre line as median, box limits as upper and lower quartiles. The notches represent confidence intervals around the median. The whiskers extend from the box limits by 1x the interquartile range. **b** Polar histogram of the number of alertness ratings as a function of time of day. **c** Alertness ratings within the first three hours after breakfast onset. Participants were instructed to rate their alertness at t = 0 min, t = 30, t = 90 and t = 150 min after breakfast start. During that ~3 hour period, they were also instructed to fast and avoid physical activity. Each black dot represents one alertness rating from one participant. The purple line shows a cubic regression of all the morning alertness ratings. Alertness immediately increased after breakfast, and then plateaued for the subsequent 2.5 hours. **d** Distribution of breakfast start time. By definition in the protocol, the first alertness rating of the day must coincide with breakfast onset. Source data are provided as a Source Data file.

In short (and see “Methods”), PREDICT1 is a prospective longitudinal study including a thousand twins and genetically unrelated adults. During the two weeks of the study, participants consumed multiple standardised breakfast meals differing in nutritional composition (Table 1), while wearing an accelerometer wristwatch and a continuous glucose monitor (CGM). Participants also recorded their food intake on a dedicated study app throughout the study, together with their alertness levels on a 0-100 visual analogue scale at several time points after the logging of each meal (Fig. 1). Demographics and descriptive statistics of the dataset are reported in Table 2 and Fig. 2.

**Table 1:** Nutritional composition of the standardised breakfast meals

Meal	Description	Energy (kcal)	Carbohydrate (g) [%E]	Fat (g) [%E]	Protein (g) [%E]	Fibre (g)
UK Average	3 muffins	502	71.2 [56.7]	22.2 [39.8]	9.6 [7.6]	2.2
Metabolic Challenge	2 muffins + 1 milkshake	890	85.5 [38.4]	52.7 [53.3]	16.1 [7.2]	2.3
OGTT	82.5 g glucose powder	300	75.0 [100]	0 [0]	0 [0]	0
High Carb	3 muffins	504	95.4 [75.7]	9.0 [16.1]	9.4 [7.5]	1.7
High Fat 1	2 muffins	500	40.5 [32.4]	34.8 [62.6]	9.0 [7.2]	1.1
High Fat 2	2 muffins	501	28.2 [22.5]	39.3 [70.6]	8.1 [6.5]	0.8
High Fibre	2 muffins + 2 fibre bars	534	94.8 [71.0]	12.9 [21.7]	9.3 [7.0]	15.3
High Protein	2 muffins + 1 milkshake	502	71.4 [56.9]	5.7 [10.2]	40.8 [32.5]	2.0

*OGTT* oral glucose tolerance test, *E* energy. The High Fat 1 and High Fat 2 meals were combined into a single High Fat meal for subsequent analysis. The “UK Average” meal consists of a medium amount of fat and carbohydrates, and is representative of the typical UK diet (NDNS survey, https://www.food.gov.uk/research/national-diet-and-nutrition-survey). The percent energy values are calculated using the approximate Atwater system (4 kcal/grams of protein, 4 kcal/grams of carbohydrates and 9 kcal/grams of fat), and thus may not necessarily sum to 100%. Adapted from^8^.

**Table 2:** Demographics and descriptive statistics of sleep and alertness

No. participants	833
Age	46.20 ± 11.93 yrs
Sex	233 M/600 F
BMI	25.83 ± 5.12 kg/m^2^
PSQI	4.86 ± 2.73
Race	747 White/33 Other or Mixed/16 Asian/7 Black
Twin status	359 NT/340 MZ/134 DZ
Country	749 UK/84 US
No. nights (total)	9,542
No. nights per Ss (grandmean)	11.45 ± 1.77
Sleep duration (grandmean)	7.66 ± 0.80 hr
Sleep efficiency (grandmean)	89.19 ± 4.22 %
Sleep onset (grandmean)	23h25 ± 0h57
Sleep offset (grandmean)	07h05 ± 0h56
No. alertness ratings	89,535
No. alertness ratings per day (grandmean)	7.94 ± 2.23
No. alertness ratings in the first three hours after breakfast (“morning”)	24,002
Alertness (all)	64.14 ± 20.25
Alertness (grandmean)	63.85 ± 13.22

Values represent mean ± standard deviation, or counts for sex, race, twin status and country. The grandmean is the average of the individual’s average across the 14 days of the study. Source data are provided as a Source Data file.

*BMI* body mass index, *PSQI* Pittsburgh Sleep Quality Index, *NT* non-twin, *MZ* monozygotic, *DZ* dizygotic, *Ss* subject.

We first tested the experimental hypothesis that day-to-day fluctuations in alertness are associated with changes in sleep the night prior, physical activity across the previous day, as well as first food intake upon awakening for the analysis day in question (targeting breakfast nutritional composition and consequential blood sugar response).

The average of all initial morning alertness ratings logged by participants within the first three hours after the start of the standardised breakfast meal was used to compute a daily morning alertness score for each participant (Fig. 2c, d). This time period was defined within the experimental design, such that participants were instructed to first rate their alertness in the morning when they started their breakfast, and then to rate their alertness at several time points during the three hours following breakfast start (t = 0, +0.5, +1.5, and +2.5 hours).

Participants were further instructed to avoid any snacking and/or physical activity during that time window (see “Methods”). This ensured a null or low impact of potential confounders (snacks, physical activity) of morning alertness, as well as a high temporal causality between the predictors of interest — prior sleep, prior physical activity, same-day morning breakfast composition and consequential glucose response, and the outcome of interest: alertness. On average, participants started their breakfast at 8h12 am ± 1h24 min (Fig. 2d). The average latency between the sleep offset (estimated from the accelerometer) and breakfast start was 1h08 min ± 1h21 min.

A linear mixed-effects model was built to test the association between each aforementioned predictor and morning alertness. A detailed description of the predictors can be found in the “Methods” section. Importantly, all predictors were included in a single linear mixed model to estimate the contribution of each predictor, whilst simultaneously adjusting for all the others. All linear mixed models reported thereafter were adjusted for age, sex, body mass index (BMI), zygosity, sunrise time, daylight saving time (DST) and weekend. To account for the natural between-person variability in sleep, the sleep predictors — duration, efficiency and waketime — were normalised using person-mean centering, i.e., expressed as a deviation from a person’s average across the two weeks of the study. Unless otherwise specified, all p-values reported in the following paragraphs were obtained from two-tailed Wald tests.

**Fig. 3:**
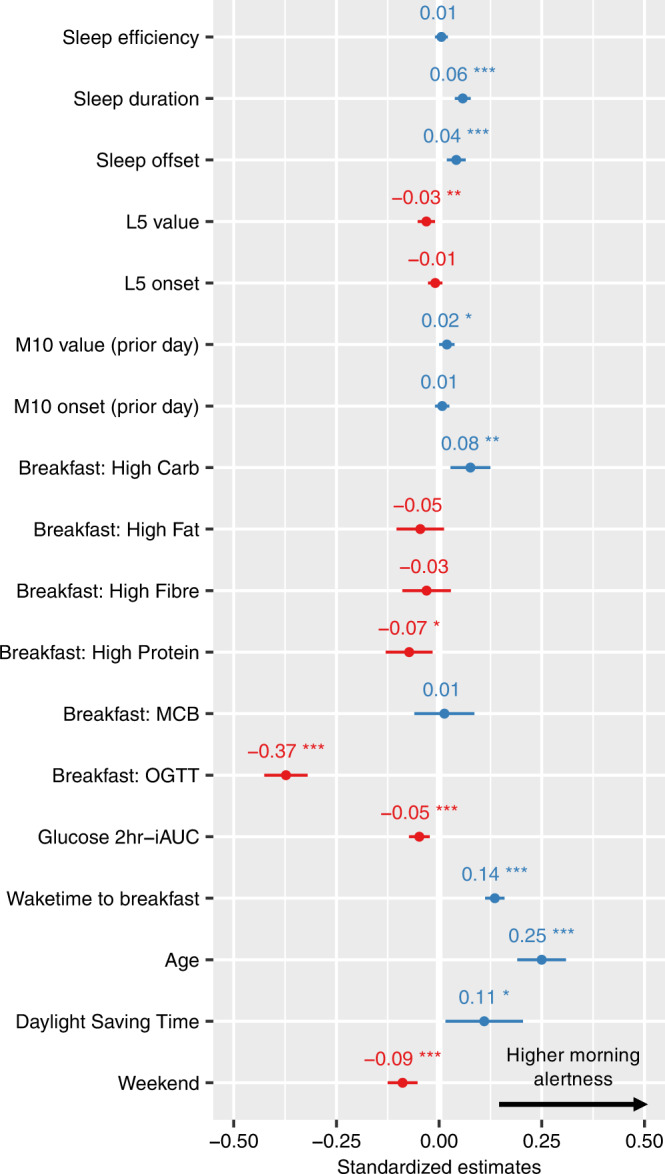
Predictors of day-to-day fluctuations in morning alertness. Standardised regression coefficients and confidence intervals from a linear mixed effect model. Sex, BMI, zygosity and sunrise time were also included in the model but are not reported here for conciseness since none of them was a significant predictor of morning alertness. Unstandardised regression coefficients and raw p-values can be found in Supplementary Table 1. Sleep predictors were normalised using person-mean centering. The dependent variable of the model is morning alertness, which is calculated by averaging the alertness ratings that were made within the first three hours after breakfast start (n = 6,744 observations). Family ID and participant ID were defined as nested random effects of the linear mixed model. Predictors with a positive coefficient (i.e predicting higher morning alertness) are shown in blue, while those with a negative coefficient (lower morning alertness) are shown in red. Error bars represent 95% confidence intervals. Stars indicate significance. P-values are based on two-tailed Wald tests (degrees of freedom = 6717) and are not adjusted for multiple comparisons. *p < 0.05, **p < 0.01, ***p < 0.001. L5 = least active 5 hours of the day, M10 = most active 10 hours of the day, MCB metabolic challenge breakfast, OGTT Oral Glucose Tolerance Test, iAUC incremental area under the curve. Source data are provided as a Source Data file.

Consistent with the hypothesis, analyses demonstrated that (1) prior sleep parameters, (2) breakfast composition and its associated post-breakfast blood glucose response, and (3) physical activity of the previous day were each significantly, and independently, predictive of morning alertness (Fig. 3 and Supplementary Table 1).

First, sleeping longer than one’s typical sleep duration was associated with higher next-morning alertness (p < 0.001). Waking up later than one’s own typical wake-up time was also associated with higher subsequent alertness, even when controlling for sleep duration (p < 0.001). A similar effect was observed for sleep onset: going to bed later than usual for a specific individual was associated with higher morning alertness (p < 0.001; Supplementary Table 2, note that sleep offset and sleep onset could not be included in the same model because of high collinearity with sleep duration). There was no significant interaction between sleep duration and sleep timing (p = 0.792; Supplementary Fig. 1). Sleep efficiency did not significantly predict morning alertness (p = 0.48). Taken together, this first set of data demonstrates that sleeping longer and/or later than typical is associated with higher next-morning alertness.

Second, the amount of physical activity that occurred across the prior day also predicted morning alertness that following day. The average acceleration value of the most active 10 hours of the previous daytime prior (the so-called M10, see “Methods”) was associated with higher alertness (p = 0.049). Conversely, the activity level in the nighttime (so-called L5, see “Methods”) was related to worse next-day morning alertness (p = 0.004). Therefore, higher levels of movement activity during the day (indicative of daytime physical movement activity), yet lower levels of physical movement activity at night, associated with more continuous and less disrupted sleep^10^, each predicted superior morning alertness.

Third, breakfast composition the morning of significantly predicted subsequent alertness (Fig. 3 and Supplementary Table 1). Specifically, the High Carbohydrates breakfast meal was associated with higher morning alertness (p = 0.002), relative to the reference standardised meal consisting of a medium amount of fat and carbohydrate (the “UK Average”, see Table 1 and Supplementary Fig. 2). By contrast, the High Protein meal was associated with lower alertness compared to the reference meal (p = 0.012). The strongest effect, however, was found for days in which participants consumed a pure glucose liquid for breakfast (i.e., Oral Glucose Tolerance Test, or OGTT, see Table 1). Here, and compared to the reference standardised meal, consumption of the OGTT was associated with marked reductions in subsequent alertness (p < 0.001). In fact, alertness following the OGTT meal was significantly lower than alertness following all the other standardised breakfast meals (Supplementary Fig. 2).

Since the OGTT is a non-standard breakfast meal, and participants were asked not to consume caffeinated drinks the morning of the OGTT (see “Methods”), the inclusion of the OGTT as a breakfast meal could potentially bias the association between alertness and the standardised meals. However, additional analyses revealed that all the above effects remained similar when removing the OGTT from the analysis while leaving other standard breakfast meals within the model (Supplementary Table 3). Moreover, caffeine intake was largely absent for the majority of observations (86% null + 12% missing = 98%), suggesting that most breakfast meals were not accompanied by any caffeinated drinks. Because of this low variance, breakfast caffeine intake was not included in the model. Nevertheless, including breakfast caffeine intake did not change the significance of the other predictors, and breakfast caffeine intake by itself was not a significant predictor of morning alertness — both with and without the OGTT (p = 0.11 and p = 0.605 respectively).

The above data describe the association between the nutritional composition of the breakfast meal consumed and subsequent morning alertness. However, such data do not address the metabolic downstream consequence of that meal: the physiological glucose response to the breakfast meal. Addressing this question, and using a CGM device (see “Methods”), post-breakfast glucose levels were uniquely associated with subsequent morning alertness, such that a lower post-breakfast glycemic load (i.e., area under the curve of blood glucose in the 2 hours following the breakfast) predicted a superior subsequent alertness (p < 0.001; Fig. 3 and Supplementary Table 1).

As a collective, analyses of this first component of the study demonstrate that morning alertness was significantly, and independently so, associated with the factors of (1) sleep (specifically a longer sleep duration, the offset timing of a later morning awakening and lower levels of movement during the night), (2) physical activity (increased activity on the previous day), (3) breakfast composition (high carbohydrates meal), and (4) post-breakfast blood glucose response (lower glycemic load).

### Predicting an individual’s baseline levels of alertness

**Fig. 4:**
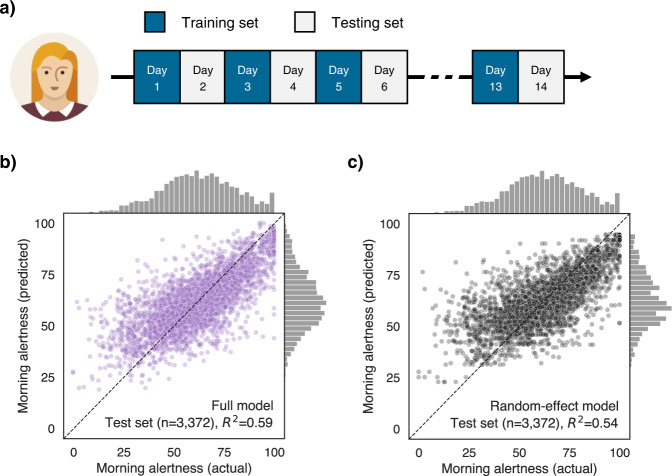
Predictions of morning alertness from the hold-out validation. **a** Hold-out validation strategy. For each participant, the multilevel model was trained on half the available days and then tested on the remaining half. Predictors of the multilevel model are shown in Fig. 3. The dependent variable of the model is morning alertness, which is calculated by averaging the alertness ratings that were made within the first three hours after breakfast start. Family ID and participant ID were defined as nested random effects of the linear mixed model. **b** Scatter plot showing the true and predicted values of morning alertness from a full model that included all the aforementioned predictors. Each dot in the scatter plot represents the morning alertness value from one day from one participant. **c** True and predicted values of morning alertness from a naive model that only included random effects. Predicted values are therefore, for each participant, the average of all the morning alertness values in the training set. Source data are provided as a Source Data file. The woman icon was downloaded from iconfinder.com.

One significant advantage of the current longitudinal design is the ability to resolve personalised, within-individual unique changes. To analytically exploit these unique, within-individual dynamics, a hold-out validation was conducted to determine whether the morning alertness of a specific given individual on a given morning was accurately predicted by the aforementioned modifiable lifestyle factors (Fig. 4a). Indeed, the model was able to explain 59% of the variance in morning alertness in the testing set (Fig. 4b). This model was then compared to a dummy model that predicted the average morning alertness for each individual across all days of the training dataset (Fig. 4c). A Bayesian model comparison revealed that the main experimental model was significantly better than the dummy model (log[Bayes Factor] =181.3), although the added gain in explained variance was relatively small (5%). The latter finding suggests a substantive consistency of morning alertness level among the same individual (i.e., low within-individual variance). Consistent with this finding, the intraclass correlation coefficient of the dummy model was 0.56, indicating that between-individual variability accounted for a larger total amount of variance in morning alertness.

As described above, that nevertheless substantial within-individual variance can be explained by the a priori collection of preceding factors  of 1) prior sleep, 2) prior physical activity, and 3) morning food composition. However, these analyses do not address the second main experimental question: what factor(s) then explain the even larger between-individual variability in levels of alertness. For example, is this set-point of daytime alertness across individuals genetically determined, and thus inherently fixed for each individual. Or rather, is this alertness set-point across the population influenced by external, modifiable trait factors (e.g. lifestyle, habitual behaviours, health)? The latter hypothesis is of particular relevance, as it would suggest that long-term targeted public-health interventions on these modifiable factors may provide a way to improve (i.e., shift up) an individual’s set-point in daytime alertness.

To test this hypothesis, the average alertness across all days of the longitudinal study, termed, “trait daytime alertness”, was calculated for each participant. A machine-learning approach then evaluated the ranked importance of trait predictors, including demographics, mental health, mood, habitual eating behaviours, as well as subjective and objective markers of sleep and physical activity. The model used a three-fold cross-validation approach coupled with a gradient boosting estimator to predict the baseline alertness of all participants based on all above predictors. Gradient boosting algorithms are optimal for this sort of task as they — unlike standard regression models — natively handle missing values in predictors and are robust to highly correlated predictors^11^. The importance rank of each predictor was then calculated on the full dataset using Shapley (SHAP) values (see “Methods”), which quantify the exact impact of a given feature — after accounting for all other features — on the predicted alertness outcome of the model.

**Fig. 5:**
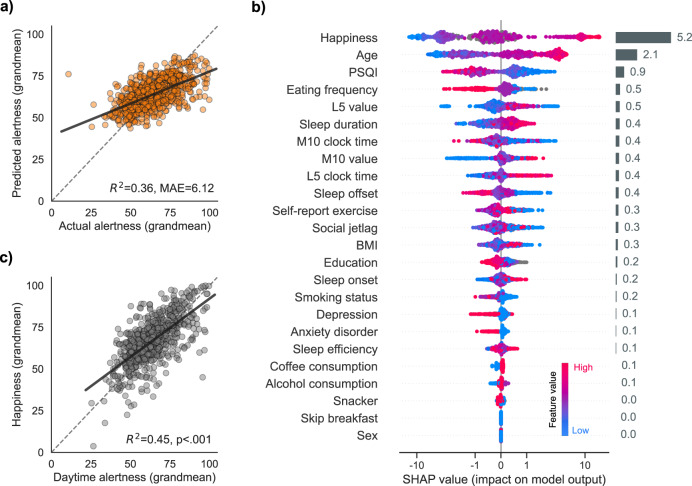
Predictors of trait alertness. **a** Cross-validated performance of the gradient boosting algorithm. Predictions of the trait alertness are plotted against the ground-truth values. Each dot represents an individual’s predicted and actual trait alertness. Trait alertness was calculated by averaging, for each individual, all their alertness rating across the two weeks of the study. **b** Features of the gradient boosting algorithm, ranked by order of descending importance. Features importance was calculated using the SHAP method (see “Methods”). Each dot on the plot is a Shapley value for a given predictor and participant. Shapley values represent, for each participant, the exact contribution of a given feature on the output of the model. The colour of the dots represents the value of a given predictor from low to high (e.g. for age, younger = blue, older = pink). Global feature importance is shown in the rightmost horizontal grey bars and was calculated by averaging the absolute Shapley values of a given predictor across all participants. **c** Trait correlation between happiness and alertness. Happiness was the most important feature of the model. Higher trait happiness is associated with higher trait alertness. Source data are provided as a Source Data file.

Consistent with the hypothesis, the machine-learning model accurately and significantly predicted what an individual’s typical level of alertness would be. Indeed, the model explained more than a third of the variance of someone’s set-point alertness in a previously unseen (cross-validated) dataset (Fig. 5a, with no statistical difference between the means of the predicted and ground-truth alertness values: paired T-test, T(832)=−0.07, p = 0.94).

The importance ranking of the predictors revealed four key top factors that had the strongest impact on the outcome of the model: (1) mood, (2) age, (3) sleep, and (4) eating frequency (Fig. 5b).

Mood, specifically  levels of daily happiness, together with the age of the individual, were the two most significant predictors of trait alertness (Fig. 5b, c), such that higher levels of happiness and increasing chronological age were each positively predictive of higher inherent levels of alertness (r = 0.67, p < 0.001 and r = 0.345, p < 0.001, respectively; Supplementary Figs. 3 and 4, with p-values adjusted for multiple comparisons using the Holm-Bonferroni method).

Interestingly, the former associations between happiness and alertness would predict that participants who suffer from mood disorders have lower levels of alertness. Post-hoc analyses afforded a test of this prediction, demonstrating that this was indeed the case. Specifically, participants with a current/former medical diagnosis of depression and/or anxiety disorder had significantly lower baseline alertness compared to the remaining participants (Welch T-test, depression: T(132)=2.855, p = 0.005; anxiety disorder: T(165)=3.24, p = 0.001; normal quantile plots (Q–Q plots) were used to check the assumption of normality; Supplementary Fig. 5).

The third key feature predicting baseline alertness was an individual’s quality of sleep. As assessed with the validated Pittsburgh Sleep Quality Index (PSQI), the higher an individual’s nightly sleep quality (reflected in lower PSQI scores), the higher their level of trait alertness (r = −0.14, p = 0.002, Fig. 5b).

The fourth top feature of predictive importance was the frequency of food intake. Here, the higher the self-reported frequency of eating (1–2 times vs. 3–4 times vs. 5+ occasions), the lower the levels of trait alertness. Indeed, participants who reported eating on average 5 times or more a day had a significantly lower baseline alertness than those who reported eating 3–4 times a day (T(731)=2.94, p = 0.01, adjusted for multiple comparisons using Tukey’s method; Q-Q plots were used to check the assumption of normality, and the Levene test was used to verify the assumption of homoscedasticity) or 1–2 times a day (T(731)=2.65, p = 0.02).

### The genetic heritability of alertness

The above findings describe a set of non-genetic, and thus modifiable lifestyle factors (with the exception of age) that are significantly associated with alertness, both in terms of day-to-day fluctuations (i.e., daily state changes) and trait level of alertness.

However, together, these factors still leave a proportion of the variance in alertness unexplained, suggesting a role for the non-modifiable factor of an individual’s genetics to prove influential. To test this last hypothesis, a standard twin model^12^ was used to test the extent of the contribution from an individual’s genetics in predicting their levels of alertness.

Broad-sense heritability was calculated using the prototypical variance component twin model approach. In short, (but see “Methods” for details), this approach decomposes the phenotypic variance into a mixture of additive and non-additive genetic factors (A and D, respectively), shared environmental factors (C), and individual-specific environmental variance plus measurement error (E). All models reported thereafter were then adjusted for age and sex.

Focusing first on the baseline levels of across the entire day alertness described in the previous section, an ACE model yielded a heritability estimate of 0.25 (95% confidence intervals [CIs] = −0.34 to 0.84), indicating a modest and non-significant contribution of genetic factors to phenotypic differences in alertness across individuals (Table 3). Consistent with our prior hypotheses and earlier results, the majority of variability in basal alertness was explained by individual-specific environmental factors (E, 57%). Model-comparison analyses revealed that trait alertness is significantly influenced by familial (i.e., A + C) factors, but not by genetic factors alone. That is, the A and C parameters could be removed individually without significantly worsening the model fit (p = 0.384 and p = 0.532), but dropping both parameters resulted in a worse model fit (p < 0.001).

**Table 3:** The heritability of alertness and sleep

		Intra-pair correlation		Genetic models				
	MZ/DZ pairs	rMZ	rDZ	${h}^{2}$ (95% CI)	A	C or D	E	p
Daytime alertness	121/38	0.43	0.30	0.25 (−0.34, 0.84)	24.9%	17.8% (C)	57.3%	<0.001
Morning alertness	120/38	0.41	0.215	0.39 (−0.26, 1.03)	38.6%	2.2% (C)	59.2%	<0.001
Happiness	88/28	0.32	0.08	0.32 (0.13, 0.51)	0%	32.0% (D)	68.0%	0.011
Sleep duration	121/38	0.51	0.22	0.51 (0.37, 0.64)	38.2%	12.5% (D)	49.4%	<0.001
Sleep efficiency	121/38	0.68	0.17	0.68 (0.58, 0.77)	0.6%	67.1% (D)	32.3%	<0.001
Sleep onset	121/38	0.60	0.305	0.595 (0.04, 1.15)	59.5%	0.7% (C)	39.8%	<0.001
Sleep offset	121/38	0.72	0.63	0.18 (−0.16, 0.51)	17.6%	54.2% (C)	28.2%	<0.001

rMZ = monozygotic intra-pair correlation coefficient, rDZ = dizygotic intra-pair correlation coefficient, ${h}^{2}$= broad-sense heritability of the phenotype, defined as the percentage of total phenotypic variance explained by genetic factors (= A in ACE models and A + D in ADE models), A = additive genetic effect, D = non-additive genetic effect, C = shared environmental effect, E = non-shared environmental effect. All models are adjusted for age and sex. Two-tailed p-values were obtained using a likelihood-ratio test comparing the goodness of fit of the full model to a restricted model that only included individual-specific environmental factors (E). Source data are provided as a Source Data file.

Second, the same analysis was conducted for trait alertness in the morning hours following breakfast consumption, rather than across the entire day. This model yielded a heritability estimate of 0.39 (best-fitting model = AE; Table 3), suggesting that alertness levels in the first hours after waking up are somewhat more influenced by genetics than alertness during the rest of the day. Once again, however, the heritability estimate were not significant (i.e., overlap with zero, −0.26 to 1.03), and individual-specific environmental factors instead explained most of the variability in trait morning alertness (59%).

Finally, and to facilitate better interpretation of the above effects still through the lens of genetics, heritability estimates were then calculated for a subset of predictors including self-report happiness and sleep parameters (Table 3). The heritability estimates of happiness and sleep offset were roughly in the same range as alertness (${h}^{2}$ = .32 and ${h}^{2}$ = .18 respectively). By contrast, other sleep parameters such as sleep duration, sleep efficiency, and sleep onset had higher heritability estimates (${h}^{2}$ ≥ 0.51), indicating a stronger contribution of genetic factors for these traits.

Overall, the heritability analyses demonstrate that an individual’s level of alertness is not strongly dependent on genetics, and reinforce the former findings that non-genetic (and thus modifiable) extrinsic factors more strongly predict differences in alertness across this population.

## Discussion

Why is it that we human beings fluctuate in our alertness from one day to the next? Why do we wake one morning feeling alert, yet another morning, flounder in that level of alertness upon awakening? The first set of analyses revealed that three key factors significantly and each independently predict how an individual awakens with alertness: 1) their prior sleep history, 2) their levels of prior physical activity, and 3) what they first eat in the morning. . Importantly, from an interventional perspective, all three of these categories are largely modifiable, and therefore represent lifestyle-realistic opportunities, or interventional levers, that may aid how an individual (and collectively, a society) awake each day, and sustain that waking alertness. We now discuss each of these factors in detail, starting with sleep.

Nights when an individual slept longer than their own typical sleep amount (rather than a standardised sleep amount), were associated with a superior (i.e., higher) degree of next-morning alertness. More than just sleep duration, however, where that sleep temporally arrived on the 24-hour clock face was also significantly associated with next-morning alertness. Specifically, sleeping later into the morning than is typical for a given individual (which in part, can give rise to longer sleep duration), predicted higher levels of alertness that following morning.

Such insights emphasise the utility of using a longitudinal study design, which allows for a definition of a person’s own individualised sleep norms, and deviations from that person-specific norm, both in sleep amount, and sleep timing. This is not simply methodological in nature or value, but further highlights the possibility that adopting a person-centric experimental approach allows for true individualised recommendations targeting more effective behavioural change to prevent failures in attentive alertness during the waking (and working) day.

A next-step challenge will be to determine the underlying mechanism(s) of how and why sleeping longer and sleeping later, relative to that unique individual’s typical norm, may transact a benefit upon morning alertness.

We offer three tenable and testable candidates. First, sleeping later in the morning means that an individual is more likely to wake up farther away from their circadian nadir, shifting toward the circadian assent. Second, sleeping later will increase the likelihood of obtaining more (or even awaken from) REM sleep^5,13,14^. Both this first and second possibilities have been associated with reduced sleep inertia^5,13^. The latter benefit upon REM sleep may be beneficially driven by higher cortical activation, or the increase in body (and brain) temperature associated with REM sleep^13,15,16^.

A third (and non-mutually exclusive) explanation for the independent benefit of sleeping longer concerns sleep pressure. Specifically, the effective discharging of sleep pressure upon awakening. This dissipation, also known as discharging of the sleep homeostat (in part, reflecting the clearance of accumulated adenosine^17^), is one of the most reliable predictors of diminished sleep inertia^4,5,18^. Indeed, long sleepers have, on average, less remaining daytime sleep pressure than short sleepers^19^. Moreover, within an individual, extending sleep duration leads to a decrease in sleep pressure^14^. In the context of our findings, sleeping longer (relative to one’s own typical sleep amount) may allow a fuller discharge of sleep pressure during the night, resulting in higher morning alertness from one day to the next.

Beyond sleep, the second main feature predicting day-to-day changes in morning alertness was the intensity of physical activity the day prior. In particular, when the extent of an individual’s physical activity was comparatively greater the day prior, individuals experienced higher levels of alertness the next morning. Covariate analyses demonstrated that this effect was not driven by a simple age-related difference in activity levels. Moreover, even though physical activity can have modest benefits upon subsequent sleep^20^, the effects we report between prior physical activity and next-day alertness remained significant when accounting for the amount of sleep that came in between. This would suggest the influence of prior physical activity and that of prior sleep upon alertness are each independent. However, a limitation of our study is that we did not quantify the electrophysiological quality of sleep, relevant considering that the effects of physical activity on subsequent sleep can include changes in the NREM quality of sleep^21^.

The final factor predicting fluctuations in next-day alertness occurred not on the day prior, or even the night prior, but the morning of. Specifically, it was the unique macronutrient composition of food that  the individual consumed for their breakfast that offered further explanatory value. Compared to a reference breakfast meal consisting of a standardised (moderate) amount of fat, carbohydrates, and protein (approximately 40/50/10% of energy, respectively), when individuals consumed a higher amount of carbohydrates (“High Carbs” breakfast), they experienced higher levels of ensuing alertness. In contrast, the “High Protein” breakfast predicted a diminished, rather than enhanced, level of alertness following sleep, relative to the reference meal. Lastly, the consumption of a pure glucose liquid bolus (oral glucose tolerance test, OGTT) was associated with a marked reduction in alertness levels−the largest drop relative to all the other standardised breakfast meals.

Importantly, however, all of the above associations for the breakfast food remained significant when postprandial blood glucose levels were adjusted for in the statistical model. Similarly, the differences in alertness between the protein and carbohydrate meals were not driven by differences in total energy content of each respective meal, since most of the breakfast meals (including the High Protein, High Carb and standardised reference) were calorically matched (i.e., isocaloric at ~500 kcal). Instead, these findings suggest that it is the actual macronutrient composition of the meal itself that contributes to the  statistically independent prediction of subsequent alertness.

That a carbohydrate-rich meal, versus a protein-rich meal, is associated with a higher level of morning alertness may seem counter to previous reports that have described either the opposite effect^22^, or no significant difference relative to protein-rich meals^23^. However, there is evidence of high carbohydrate intake linked to superior alertness levels, consistent with our findings^24^. In addition,   increasing protein intake in drosophila models was correlated with decreased postprandial alertness^25^, suggesting that a meal lower in protein may be optimal for increasing alertness.

Beyond the effects of the high carbohydrate meal itself, one of the strongest effects revealed in the current study was the marked reduction in alertness on days when participants consumed a high-sugar amount, here controlled using a standardised liquid glucose bolus at breakfast (the oral glucose tolerance test (OGTT)). The OGTT consists of 100% monosaccharide glucose. That a large dose of glucose predicts a drop in alertness may appear contradictory to the aforementioned association of the High Carb breakfast linked to an increase in alertness. However, despite their common high carbohydrate content, there are three key differences between the High Carb and OGTT meals that aid in resolving this empirical tension.

First, unlike the OGTT, the High Carb breakfast a contained 23% proportion of other macronutrients, of which 16% was fat and 7% was protein. The ratio of carbohydrates-to-protein is known to modulate tryptophan availability in the brain and thus serotonin synthesis^26^. Specifically, pure  carbohydrate (i.e., a ratio of 1:0, as seen in the OGTT) may drive the strongest sedative effect through a surge in serotonin synthesis and thus brain-available tryptophan associated with impaired alertness^27^. Our finding would therefore suggest that the combination of other macronutrients (e.g., protein, fat, fibre) paired with carbohydrate determines the true synergistic impact of the meal on subsequent alertness.

The second key distinction between the High Carb meal and OGTT meal is the sugar form. The OGTT consists exclusively of glucose, whereas the High Carb meal consists of sucrose, which is composed of one molecule of glucose, and one molecule of fructose. Unlike glucose in the OGTT, the fructose represented in the High Carb meal results in a more modest effect on blood glucose and circulating insulin levels^28^. Therefore, the High Carb meal can induce a lower glycemic response than the OGTT^29^. This strong surge in blood glucose levels caused by the OGTT may therefore result in an inhibition of the wake-promoting neurons in the hypothalamus^30^, and this loss of hypothalamic drive may ultimately lead to reduced alertness.

The third non-mutually exclusive explanation for why the pure glucose meal significantly impaired alertness concerns the lower total caloric content that the OGTT represented, relative to  the other standardised meals in the study (300 kcal vs ~500 kcal). Within this mechanistic framework, the caloric deficit would result in post-consumption hunger^31^, the effects of which have been associated with  lower levels of general alertness^32^.

The macronutrient composition of a meal can significantly dictate the subsequent change in blood glucose^9^. However, different individuals respond with markedly different blood glucose responses to the same macronutrient meal due to a broad collection of reasons^9^. This suggests that, independent of the nutritional content of the food an individual eats, there is a need to additionally measure, and account for, the inter-individual difference in blood glucose response to that meal.

Guided by this dissociation, the final analyses assessed associations between alertness and the body’s glycemic food response Here, on days when an individual experienced a higher blood glucose response to that same standardised breakfast meal, the lower their levels of alertness that following morning, and vice versa. Importantly, this effect was not explained by breakfast macronutrient composition. Therefore, our findings establish that it is both the macronutrient profile of food and the way in which the body processes that food — here on the basis of glycemic blood glucose levels. We show that both independently predict that relationship with morning alertness.

That a higher level of blood glucose is associated with a lower level of alertness would, after first glance, contravene the popular cultural belief of a “sugar rush” that boosts vigilance. However, contrary to this common fiction, and consistent with the current findings, experimental evidence has established that high glucose consumption results in a “sugar crash” and thus reduced alertness.  Specifically, attentive levels of consciousness and associated cognition decrease soon after the ingestion of high-glycemic foods, such as potatoes and sugar-sweetened beverages^27,33^. Adding further support to the notion of a sugar crash demonstrated in the current study, a recent meta-analysis reported that high subsequent glycemic responses to morning breakfast meals are associated with impaired, rather than enhanced, cognitive performance^34^.

How and why alertness levels drop significantly following a high-glycemic meal has been linked to glucose-sensing neurons in the brain and may accurately account for our related findings. Indeed, when glucose-sensing neurons are activated by high(er) levels of blood sugar^30,35,36^, these sugar-sensing brain cells inhibit the otherwise wake-promoting orexin system within the lateral hypothalamus, the blockade of which results in decreased alertness^30^.

Beyond empirical and mechanisms insights, the current study may aid more broadly in the development of behavioural recommendations at a public-health or government level. Perhaps most applicable, the current results suggest that avoiding high-glycemic-response breakfast is associated with optimal alertness throughout the morning. This may be especially germane in the context of education, where alertness is essential for effective knowledge acquisition in the classroom. In fact, this issue is particularly noteworthy considering the rapidly growing trend for teenagers and young adults to consume sugar-sweetened energy drinks as an alternative to a whole-foods breakfast^37^—a trend that would only serve to increase sleepiness in morning classes.

Findings from our first experimental question addressed the factors that account for fluctuations in alertness within an individual, from one day to the next. However, there was also large and substantial between-person variability in average levels of alertness. Our  second experimental question sought to explain why this is the case i.e., why do some individuals have a higher level of overall alertness “set-point” common across all days?

A prominent genetic influence would at first seem logical. However, twin-pair analyses indicated that the genetic contribution in daytime alertness was quantifiably small overall, with a heritability estimate of 0.25, demonstrating that most of the trait variation in alertness comes from non-genetically shared, individual-specific factors.

Our additional machine-learning analysis further revealed that it is non-genetic factors which best account for differences in alertness set points across individuals. Specifically, three independently contributing components were revealed that most significantly predicted trait levels of daytime alertness: (1) positive mood (specifically self-report happiness), (2) age, and (3) an individual’s self-reported sleep quality.

The feature of positive mood, and specifically levels of happiness, was the strongest predictor of general alertness across individuals, such that the happier an individual was, the higher their baseline levels of alertness. Two tenable, non-mutually exclusive, explanations may explain this association. First, the psychological state of happy mood is accompanied by an aroused autonomic state during wakefulness^38^. This increased autonomic activation may therefore in turn promote cognitive arousal and alertness. Second, there is a well-established bidirectional association between happiness and serotonin^39^; a neurotransmitter that has a key role in the modulation of alertness^40^. Higher positive mood may thus be associated with higher levels of brain serotonin, and consequently higher activity in wakefulness-promoting brain regions.

Interestingly, of the varied sleep measures assessed, it was subjective sleep quality, rather than any objective sleep metrics, that ranked as a more important predictor of an individual’s trait alertness. This association with between-person differences in alertness adds to a rapidly emerging set of data suggesting that sleep quality, as much if not more than, sleep quantity, most accurately explains the variability in sleep-dependent brain and body outcomes, including mental health and well-being^41,42^, sustained attention^43^, cardiovascular health^44^, and all-cause mortality^45^.

The current findings must be appreciated within the context of a number of important limitations. First, alertness was measured subjectively (visual analogue scale), and should therefore be interpreted in light of the potential biases associated with self-report methods. That said, subjective alertness ratings correlate significantly with objective EEG spectral power activity indexing homeostatic sleep pressure, or sleepiness^46–48^, suggesting some level of objective validity. Second, though the population cohort assessed in this study was composed of healthy individuals, we did not formally screen for sleep-disordered breathing. Therefore, we cannot exclude the possibility that a sub-group of participants existed that may have had sleep apnea, which could have influenced the results. Third, no sleep logs were collected in this study, with sleep/wake detection relying on accelerometry, which has been validated against gold-standard polysomnography in both healthy individuals and patients with sleep disorders (mean concordance statistic of 0.86 and 0.83, respectively)^49^. Fourth, light exposure in the first hours of the morning was not measured in this study. Bright light exposure has been shown to increase the cortisol awakening response^50^ and increase daytime alertness^51^, and may thus have offered additional explanatory insights in predicting morning alertness. Fifth, that the standardised breakfast meals were associated with morning alertness differently based on their macronutrient composition should not be used solely to derive conclusions on recommending absolute quantities of a single macronutrient (e.g. the simplistic notion that consuming more grams of carbohydrates will increase alertness). Indeed, every meal in the study (with the exception of the OGTT) included some amount of each of the major macronutrients, and the meals were therefore not completely separable in their macronutrient composition. Last, despite the large sample size of our study, one cannot exclude a potential selection bias. Specifically, participants in PREDICT1 study were of robust health (see inclusion criteria), and therefore may not be representative of a pure random sample of the US and UK population.

In summary, we demonstrate  that fluctuations in morning alertness within an individual from one day to the next are significantly and independently predicted by four modifiable factors of (1) sleep the night before, (2) higher physical activity on the day prior, (3) a breakfast rich in carbohydrates, and (4) a lower glycemic response in the hours following breakfast consumption. Beyond an explanation of day-to-day fluctuations in alertness, and counter to a strong trait determinant model, genetics offered a modest influence upon an individual’s set-point of alertness. Instead, trait levels of alertness across individuals were best predicted by their level of positive mood, their age, and their self-reported sleep quality.

More broadly, our results reveal a set of key factors associated with alertness that are, for the most part, not fixed. Instead, the majority of factors associated with alertness are modifiable, and therefore permissive to behavioural intervention. Such findings may help inform public health recommendations towards reducing the non-trivial mortality, financial and societal burden caused by insufficient alertness.

## Methods

### Study design and participants

We sought to test our hypothesis and predictions using the Personalized Responses to Dietary Composition Trial (or “PREDICT1”). PREDICT1 is a single-arm, single-blind intervention study, whose overall objective is to understand glucose, insulin, lipid and other postprandial responses to foods based on the individual’s characteristics, including molecular biomarkers, lifestyle factors, in combination with the nutritional composition of the food. The official start and end dates for the study were 5 June 2018 and 8 May 2019, the first participant was enrolled on 4 August 2018 and the last clinical visit was completed on 24 April 2019, with the primary cohort based at King’s College London in the UK and a second cohort (that underwent the same profiling as in the UK) assessed at Massachusetts General Hospital in Boston, MA, USA. In the UK, participants (target enrollment, 1,000 participants) were recruited from the TwinsUK cohort, a prospective cohort study, and online advertising. In the USA, participants (target enrollment, 100 participants) were recruited through online advertising and research participant databases. The written informed consent and ethical committee approvals covered all analysis reported in the current study in addition to the key primary outcomes described in^9^. The trial was registered on ClinicalTrials.gov (registration number: NCT03479866, first posted on March 27, 2018) as part of the registration for the PREDICT program of research, which also includes two other study protocol cohorts (not analysed in the current study). The trial was run in accordance with the Declaration of Helsinki and Good Clinical Practice. The study was approved in the UK by the Research Ethics Committee and Integrated Application System (IRAS 236407) and in the US by the Institutional Review Board of Partners Healthcare (Protocol # 2018P002078). Participants did not receive financial compensation for taking part in the study.

Study participants were healthy individuals aged 18–65 years, who were able to provide written informed consent. Exclusion criteria included ongoing inflammatory disease; cancer in the last three years (excluding skin cancer); long-term gastrointestinal disorders including irritable bowel disease or Celiac disease (gluten allergy), but not including irritable bowel syndrome; taking immunosuppressants or antibiotics as daily medication within the last three months; capillary glucose level of >12 mmol l–1 (or 216 mg dl–1), or type 1 diabetes mellitus, or taking medication for type 2 diabetes mellitus; currently experiencing acute clinically diagnosed depression; heart attack (myocardial infarction) or stroke in the last 6 months; pregnancy; and vegan or experiencing an eating disorder or unwilling to consume foods that are part of the study. Diagnosis or symptoms of any sleep disorders, circadian rhythm disorders, or neurocognitive disorders were not exclusionary. Similarly, the use of medication to impact sleep, circadian or brain function was not exclusionary.

A total of 970 generally healthy adults from the United Kingdom (including non-twins, monozygotic [MZ] twins and dizygotic [DZ] twins) as well as 95 healthy adults from the United States (all non-twins) were enrolled and completed baseline clinic measurements, as well as a two-week at-home phase. For more details on the clinic measurements, we refer the reader to the online protocol^8^.

During the study’s home-phase, participants consumed multiple standardised test meals differing in macronutrient composition (carbohydrate, fat, protein and fibre), while wearing a physical activity monitor. Standardised meals were consumed at breakfast during the first 9-11 days of the home period, and additionally for lunch on the two first days. Participants recorded their dietary intake and alertness on the Zoe study app throughout the study. Following completion of the home-phase, participants returned all study samples and devices to study staff via standard mail.

### Data collection and analysis

For an exhaustive description of all the outcomes measured in the PREDICT1 study, we refer the reader to the full online protocol^8^. Briefly, key outcomes included postprandial metabolic responses (blood triglyceride, glucose, and insulin concentrations) to sequential mixed-nutrient dietary challenges administered in a tightly controlled clinical setting on day 1. A second set of outcomes was assessed over the subsequent 13 d at-home period. Primary outcomes include gut microbiome profile, blood lipids and glucose, sleep, physical activity, and hunger and appetite assessment. The main analysis of the primary outcomes has been reported elsewhere^8,52^. The current study reports a non-preregistered/exploratory analysis of the association between the secondary outcome of subjective alertness and the primary outcomes of sleep, physical activity, diet, and blood glucose (all measured during the at-home phase of the study). Data from the UK and US sub-cohorts were combined into a single dataset in the current study.

#### Questionnaires

Data on socio-demographic characteristics, medical health, habitual diet, and lifestyle were collected via a self-administered baseline questionnaire during the clinic visit. *Education*. Academic education was measured on a scale from 0 (no qualifications) to 8 (postgraduate degree). *Subjective sleep*. Subjective sleep quality was assessed using the well-validated Pittsburgh Sleep Quality Index (PSQI)^53^. The PSQI measures 7 domains of sleep quality over the past month to provide a global score (0-21) of overall sleep quality, with higher scores indicating poorer sleep quality. In addition, participants were asked to report their typical bedtime and waketime, in both weekdays and weekends. The absolute difference between the midpoint of sleep in weekdays and weekends was then used to quantify social jetlag^54^, with higher values indicating a greater mismatch between an individual’s own biological rhythm and the daily timing determined by social constraints (assuming that most individuals worked during the weekdays and did not work during the weekend). *Habitual diet and eating behaviours*. Habitual diet was measured using the European Prospective Investigation into Cancer and Nutrition (EPIC) food frequency questionnaire (FFQ) in the UK cohort and the Harvard semi-quantitative FFQ in the US cohort. The questionnaire also included questions related to eating frequency (“how many times do you eat in a day?”), habitual coffee and alcohol consumption, and whether the participants usually skip breakfast. *Exercise*. Self-report exercise frequency was measured with the following question: “In the past year, how frequently have you typically engaged in physical exercises that raise your heart rate and last for 20 minutes at a time?” *Mental health*. Current/former clinical diagnosis of depression and anxiety disorder was measured using the following questions: *“Has a doctor ever told you that you have/had any of the following conditions? [clinical depression, anxiety or stress disorder]”*.

#### Standardised test meals

Upon completion of their baseline visit, participants received a home-phase meal pack containing test-meals varying in macronutrient composition, which they consumed according to standardised instructions for breakfast and, on some days, lunch. Test meals consisted of either an oral glucose tolerance test (OGTT) or muffins, which were consumed on their own or paired with chocolate milk, a protein shake or commercial fibre bars. The description and nutritional composition of the test meals can be found in Table 1. Test meals were consumed in a different order depending on which protocol group participants were assigned to, as described in the online protocol^8^. Test meals were prepared and packaged in the Dietetics Kitchen (Department of Nutritional Sciences, King’s College London). As is common practice for postprandial studies^55^, meal sizes were similar across all participants and not normalised by weight or total daily energy expenditure.

Participants were instructed to fast for a minimum of 8 hours prior to consuming a test breakfast meal (i.e., avoid nighttime snacking), and to fast for 3 or 4 hours after the test meal consumption. Furthermore, they were advised to limit exercise and drink only plain, still water during the fasting periods. When fasting was completed, participants could eat, drink and exercise as they liked for the rest of the day. Participants were asked to consume all muffin-based meals within 10 minutes and the OGTT within 5 minutes, and to notify study staff if this was not achieved, in which case the data were excluded from the analysis.

If the participant chose to accompany their home-phase muffin-based test meals with a tea or coffee (with up to 40 ml of 0.1% fat cow’s milk, but without any sugar or sweeteners), they were instructed to consume this drink consistently, in the same strength and amount, alongside all muffin-based test meals throughout the study. Participants were instructed to not consume any food or drink other than water alongside the OGTT. They recorded test meals and any other dietary intake within fasting periods, including accompanying drinks in the study app (see next section) with the exact time of consumption and ingredient quantities so that study staff could monitor compliance. Only test meals that were completed according to instructions were included in the analysis.

#### Food and alertness logging via mobile study app

The Zoe study app was developed to support the PREDICT 1 study by serving as an electronic notebook for study tasks. The app sent participants notifications and reminders to complete tasks at certain time-points, such as when their test lunch meals were due. Participants logged their full dietary intake using the study app over the 14-day study period, including all standardised test meals and free-living foods, beverages (including water) and medications.

Data logged into the app was uploaded onto a digital dashboard in real time and reviewed and assessed for logging accuracy and study guideline compliance by study staff. The Zoe study app contained a database of generic and branded food items with nutritional information sourced from generic data sources, commercial food databases under licence, back-of-pack information from commercial providers, and publicly available restaurant nutritional data. It also allowed participants to photograph back of pack labels in cases where this information was missing from the nutritional database, and where possible, the photographed information was entered into the database by study staff.

The app also prompted participants to report their alertness levels on a visual analogue scale^56^ by displaying the question “how alert are you?”. These app notifications appeared at t = 0 (time of logging) and regular intervals (+0.5, +1.5, +2.5 hours) following the logging of a breakfast, lunch or dinner meal. However, given the free-living conditions of the study, participants did occasionally miss one or more ratings, resulting in a variable number of alertness ratings per day per participant. The app also prompted participants to report their happiness and anxiousness levels once per day at ~9 PM local time. Here again, a visual analogue scale was used with the following question: “How have you been feeling generally over the whole day: How happy have you felt”?

For both the meals and alertness data, participants with less than 5 days of valid data were removed (n = 56 [5.3%] and n = 42 [3.49%] participants excluded, respectively). Second, participants that travelled in a different timezone during the two weeks of the home-based study were also excluded (n = 65 [5.59%] participants). Data regarding travel to a different time zone in the weeks before the study was not available.

#### Postprandial glucose

Interstitial glucose was measured every 15 min using Freestyle Libre Pro CGMs (Abbott). Monitors were fitted by trained nurses on the upper, non-dominant arm at participants’ baseline visit and were covered with Opsite Flexifix adhesive film (Smith & Nephew Medical) for improved durability, and were worn for the entire study duration. The 2 hours incremental area under the curve (2hr-iAUC) was used for analysis of the postprandial glucose response^9^. The distribution of glucose 2hr-iAUC was skewed and the data was thus transformed using a Yeo-Johnson power transformation.

#### Sleep/wake

Sleep/wake patterns were measured using a triaxial accelerometer (AX3, Axivity, Newcastle Upon Tyne, UK). The accelerometer was fitted by clinical practitioners at the baseline clinic visit on the non-dominant wrist and worn for the duration of the study (except during water-based activities, including showers and swimming), after which they were removed on day 15 and mailed back to study staff. The accelerometer was programmed to measure acceleration at 50 Hz with a dynamic range of ±8 *g* (where *g* refers to the standard acceleration of gravity, i.e., approximately 9.81 m/s^2^). Non-wear periods were defined as windows of at least 1 hour with less than 13 m*g* for at least 2 out of 3 axes, or where 2 out of 3 axes measured less than 50 m*g*^57^.

Raw accelerometer data was analyzed using the “GGIR” R package version 1.10-7^58^. Sleep/wake detection was then quantified using the validated algorithm described in ref. 49, which uses the variance in the accelerometer z-axis angle together with a set of heuristic rules to determine sleep periods. This algorithm does not require a sleep diary and has been validated against gold-standard polysomnography in both healthy individuals and patients with sleep disorders, with a mean concordance statistic of 0.86 and 0.83, respectively^49^. For each night and each participant, the following sleep metrics were calculated (see Fig. 1): sleep onset, sleep midpoint, sleep offset, sleep duration (defined as the elapsed time from sleep onset through sleep offset, or sleep period time [SPT]), wake after sleep onset (WASO), total sleep time (TST; = SPT - WASO), sleep efficiency (SE, = TST / SPT). SE was calculated using the SPT and not the more common total time in bed as denominator because the absence of sleep diary data precludes the accurate estimation of bedtime prior to sleep. For the same reason, the algorithm is unable to characterise sleep onset latency (the time between going to bed and falling asleep). The GGIR algorithm is not able to detect naps and therefore only nighttime sleep parameters were included in subsequent analyses.

A set of thresholds was then applied to remove invalid nights or participants, consistent with typical practices^59^. First, any nights with a TST outside the range of 2 to 15 hours, or a SE below 20%, was excluded (376 nights, 2.5%). Second, nights with more than 10% classified as invalid were excluded (403 nights, 2.65%). Third, nights with a sleep onset between 8 AM and 5 PM or a sleep offset after 12 PM were excluded (45 nights, 0.3%). Finally, participants with less than 5 days of valid sleep data (n = 60, 5.53%) were removed, consistent with the preprocessing of the food and alertness data.

#### Physical activity

Physical activity was measured using the accelerometer and features were calculated, for each day and each participant, using the GGIR software. Specifically, these features consisted of the M10 and L5 values, and their associated onset timings. M10 and L5 refer to the most-active 10 hours and the least-active five hours of each day, respectively, and are commonly studied measures relating to circadian activity^10,59^. The M10 was defined as the 10-h period with the maximum average acceleration, estimated using a 10 hours moving average. The L5 was defined as the 5-h period with the minimum average acceleration, estimated with a 5-hours moving average. For these two metrics, the onset timings was also calculated, defined as the number of hours elapsed from the previous midnight. Once again, participants with less than 5 days of valid physical activity data were removed (n = 51, 4.72%).

#### Data concatenation

Sleep, meal (including postprandial glucose), physical activity and alertness data were all merged into a single dataframe to facilitate statistical analyses. Here, a strict inner-merge was performed, meaning that only the participants and days with valid food, physical activity, alertness and sleep data were included in the final dataframe. The sleep and physical activity features were shifted by one day to ensure a valid temporal directionality, i.e., that sleep/physical activity occurred before, and not after, the alertness outcome.

A final quality check was applied that consisted of removing, for each day and each participant, the alertness ratings that were logged one hour or more before the algorithm’s predicted sleep offset (916 ratings, 1.0%). Concretely, this removes the alertness ratings that were input before sleep and after midnight (as these should technically be counted for the previous day).

### Statistical analyses

#### Multilevel modelling

Linear mixed-effects models were used to measure the statistical association of sleep, food intake and its associated glucose response, and physical activity with subsequent alertness. Unless specified otherwise, all multilevel models were adjusted for age, sex, body mass index (BMI), twin status (MZ, DZ or NT), sunrise time, daylight saving time (DST), and weekend (i.e., whether the day on which the person wakes up is a Saturday or Sunday); with family identifier and subject identifier defined as nested random effects.

Income, work schedule (including shift work) and household size were not available in PREDICT1 and therefore the statistical models could not be adjusted for those.

To account for the natural between-person variability in sleep, the sleep predictors — duration, efficiency and waketime — were normalised using person-mean centering. That is, they were expressed, for each participant separately, as a deviation from this individual’s average calculated across the two weeks of the study.

All multilevel analyses were performed in R^60^ using the “lme4”, “lmerTest”,“sjPlot” and “emmeans” packages^61–64^. Goodness-of-fit was evaluated with the conditional ${R}^{2}$^65^. For all multilevel models, the variance inflation factor (VIF) was used to check for multicollinearity. When multicollinearity was detected (VIF > 5), the correlated predictors were removed from the model and/or split into two separate multilevel models^66^. Diagnostic plots were used to assess the validity of the fitted models. For each multilevel model, these included scatterplots of standardised residuals by fitted values and observed versus fitted values. Normal quantile plots (Q–Q plots) were used to check the assumption of normality of the residuals and random effects.

The performance of the multilevel model in predicting new, unseen data was then tested using a hold-out validation. This step is increasingly recommended to prevent overfitting and improve the interpretability of the findings^67^. The dataset was separated into a training and testing set, based on a split of odd and even days (e.g. training = days 1, 3, 5, …; testing = days 2, 4, 6, …). Importantly, the assignment of odd days to the training or testing set was randomly decided for each participant. Then, a multilevel model was fitted on the training set, using the same predictors and random effects as the main model. The resulting regression coefficients were then used to make predictions on the testing set. Performance was evaluated using the coefficient of determination (r-squared) between the true and predicted alertness values. All statistical tests reported in the manuscript are two-tailed.

#### Machine-learning analysis of the trait predictors of alertness

For between-person (i.e., trait) analysis of alertness, a machine-learning approach was used to evaluate the relative importance of a large number of trait measures on alertness. These predictors included: the age, sex, education level, smoking status, BMI, average sleep and physical activity parameters calculated across the two weeks of the study, self-report measures of subjective sleep quality (PSQI score) and social jetlag, self-report happiness, self-report habitual amount of exercise, self-report medical diagnosis of depression or anxiety disorder, and self-report eating behaviours — including whether or not the participant usually skip breakfast, habitual coffee and alcohol consumption, eating frequency and snacking.

Several of these parameters were highly correlated and/or contained missing values. For these two reasons, the association of these predictors with alertness could not be evaluated using a standard regression approach, which would have resulted in a dramatic decrease in sample size as well as invalid regression coefficients because of multicollinearity. Addressing these issues, a gradient boosting machine-learning algorithm (LightGBM,^11^) was used as the primary analytical method for the between-person analysis. Gradient boosting algorithms are based on decision trees and are therefore robust to multicollinearity in predictors. In addition, they natively support missing values, without the need for deletion or imputation. The LightGBM model was trained with 50 estimators and a random subsampling of all features and samples (50%) before building each tree. Performed of the model on unseen data was evaluated using a 3-fold cross-validation of the full dataset.

Next, Shapley values were used to assess the unique contribution of each feature in predicting trait alertness. Shapley values have several desirable properties that make them ideal to evaluate the unbiased feature importance of the predictors of a statistical model. Specifically, they quantify, for each observation (i.e., participant), the exact impact of a given feature — after accounting for all other features — on the outcome of the model. Shapley values were first computed for each feature and each participant using the SHAP library^68^. Global feature importance was then calculated by averaging the absolute Shapley values of a given predictor across all observations.

All machine-learning algorithms were conducted in Python using the “scikit-learn”, “lightgbm“, “shap” and “pingouin” packages^11,68,69^.

#### Heritability analyses

A large proportion of the main cohort consisted of pairs of identical (MZ) and fraternal (DZ) twins, which allows a test of genetic influences upon alertness and sleep inertia. For each dependent variable, we first calculated the intra-pair correlation separately for MZ and DZ siblings. The former share the vast majority of their germline DNA sequence^70^, while the latter are assumed to share on average 50% of their segregating genetic material. DZ twins are, however, presumed to share their common environmental influences (e.g. family) to the same extent as MZ twins. Therefore, the degree to which MZ siblings have a higher correlation for a specific trait than DZ siblings reflects the extent of genetic influence on this trait.

Heritability was then calculated using a standard twin model^71^, which decomposes the observed phenotypic variation into a combination of additive (A) and non-additive (D) genetic variance, common environmental variance (C; familial influences that contribute to twin similarity) and individual-specific environmental variance plus measurement error (E). The combination of these factors that best matches the observed data is found with structural equation modelling techniques. Because the C and D factors are negatively confounded, they cannot be estimated simultaneously. Therefore, following standard guidelines, an ACE model was used when the DZ twin correlation was more than half the MZ twin correlation, and an ADE model otherwise. The broad heritability (${h}^{2}$) was then defined as the percentage of total phenotypic variance that could be explained by genetic factors (= A in ACE models and A + D in ADE models).

The significance of genetic factors (A and/or D) was assessed by means of likelihood ratio tests comparing the full model with a nested model in which these factors were constrained to be zero. When the fit significantly worsened, the contribution of genetic factors was considered significant. Finally, the Akaike Information Criterion (AIC) was used to determine the best-fitting model, with lower AIC indicating a better fit of the model to the observed data.

All heritability analyses were conducted using the “mets” R package^72^. Twin models were adjusted for age and sex. To account for repeated measurements in the twin models, analyses focused on the participants’ grand-averaged values^73^.

### Reporting summary

Further information on research design is available in the Nature Portfolio Reporting Summary linked to this article.

## Supplementary information

Supplementary Information

Reporting Summary

## Data Availability

The data of the at-home phase of the PREDICT1 trial, which supports the findings of this study, are held by Zoe Ltd. These data were used under license for the current study and are therefore not publicly available. Data are however available from the authors upon reasonable request and with permission of Zoe Ltd. The data of the baseline in-clinic visit of the PREDICT1 trial are held by the department of Twin Research at King’s College London. The data can be released to bona fide researchers using normal procedures overseen by the Wellcome Trust and its guidelines as part of our core funding. The application can be found at: https://twinsuk.ac.uk/resources-for-researchers/access-our-data/. Data must be anonymized and conform to General Data Protection Regulation standards. Source data are provided with this paper.

## Source data

Source data

## Code availability

All scripts used to run the analysis are available from the authors upon request.

## References

[1] Tefft, B. C. & Others. *Prevalence of motor vehicle crashes involving drowsy drivers, United States, 2009-2013*. (Citeseer, 2014). 10.1016/j.aap.2011.05.028 22269499

[2] Rakel RE (2009). Clinical and societal consequences of obstructive sleep apnea and excessive daytime sleepiness. Postgrad. Med..

[3] Hafner M, Stepanek M, Taylor J, Troxel WM, van Stolk C (2017). Why sleep matters-the economic costs of insufficient sleep: a cross-country comparative analysis. Rand Health Q.

[4] Vallat R, Meunier D, Nicolas A, Ruby P (2019). Hard to wake up? The cerebral correlates of sleep inertia assessed using combined behavioral, EEG and fMRI measures. Neuroimage.

[5] Hilditch CJ, McHill AW (2019). Sleep inertia: current insights. Nat. Sci. Sleep..

[6] Spaeth AM, Goel N, Dinges DF (2014). Cumulative neurobehavioral and physiological effects of chronic caffeine intake: individual differences and implications for the use of caffeinated energy products. Nutr. Rev..

[7] Trotti LM (2017). Waking up is the hardest thing I do all day: Sleep inertia and sleep drunkenness. Sleep. Med. Rev..

[8] Berry, S. et al. Personalised REsponses to DIetary Composition Trial (PREDICT): an intervention study to determine inter-individual differences in postprandial response to foods. *Protocol Exchange*10.21203/rs.2.20798/v1 (2020).

[9] Berry, S. E. et al. Human postprandial responses to food and potential for precision nutrition. *Nat. Med*. 10.1038/s41591-020-0934-0 (2020). 10.1038/s41591-020-0934-0 PMC8265154 32528151

[10] Gonçalves BSB, Adamowicz T, Louzada FM, Moreno CR, Araujo JF (2015). A fresh look at the use of nonparametric analysis in actimetry. Sleep. Med. Rev..

[11] Ke, G. et al. LightGBM: A Highly Efficient Gradient Boosting Decision Tree. in *Advances in Neural Information Processing Systems 30* (eds. Guyon, I. et al.) 3146–3154 (Curran Associates, Inc., 2017).

[12] Polderman TJC (2015). Meta-analysis of the heritability of human traits based on fifty years of twin studies. Nat. Genet..

[13] Silva EJ, Duffy JF (2008). Sleep inertia varies with circadian phase and sleep stage in older adults. Behav. Neurosci..

[14] Skorucak, J., Arbon, E. L., Dijk, D.-J. & Achermann, P. Response to chronic sleep restriction, extension, and subsequent total sleep deprivation in humans: adaptation or preserved sleep homeostasis? *Sleep* 41, (2018). 10.1093/sleep/zsy078 29722893

[15] Harding EC, Franks NP, Wisden W (2020). Sleep and thermoregulation. Curr. Opin. Physiol..

[16] Wamsley EJ, Hirota Y, Tucker MA, Smith MR, Antrobus JS (2007). Circadian and ultradian influences on dreaming: a dual rhythm model. Brain Res. Bull..

[17] Porkka-Heiskanen T, Kalinchuk AV (2011). Adenosine, energy metabolism and sleep homeostasis. Sleep. Med. Rev..

[18] Marzano C, Ferrara M, Moroni F, De Gennaro L (2011). Electroencephalographic sleep inertia of the awakening brain. Neuroscience.

[19] Aeschbach D (2001). Evidence from the waking electroencephalogram that short sleepers live under higher homeostatic sleep pressure than long sleepers. Neuroscience.

[20] Kredlow MA, Capozzoli MC, Hearon BA, Calkins AW, Otto MW (2015). The effects of physical activity on sleep: a meta-analytic review. J. Behav. Med..

[21] Park I (2021). Exercise improves the quality of slow-wave sleep by increasing slow-wave stability. Sci. Rep..

[22] Zeng Y-C (2011). Influences of protein to energy ratios in breakfast on mood, alertness and attention in the healthy undergraduate students. Health.

[23] Boelsma E, Brink EJ, Stafleu A, Hendriks HFJ (2010). Measures of postprandial wellness after single intake of two protein-carbohydrate meals. Appetite.

[24] Sihvola N (2013). Breakfast high in whey protein or carbohydrates improves coping with workload in healthy subjects. Br. J. Nutr..

[25] Murphy, K. R. et al. Postprandial sleep mechanics in Drosophila. *Elife* **5**, e19334 (2016). 10.7554/eLife.19334 PMC5119887 27873574

[26] Wurtman RJ (2003). Effects of normal meals rich in carbohydrates or proteins on plasma tryptophan and tyrosine ratios. Am. J. Clin. Nutr..

[27] Mantantzis K, Schlaghecken F, Sünram-Lea SI, Maylor EA (2019). Sugar rush or sugar crash? A meta-analysis of carbohydrate effects on mood. Neurosci. Biobehav. Rev..

[28] Merino, B., Fernández-Díaz, C. M., Cózar-Castellano, I. & Perdomo, G. Intestinal Fructose and Glucose Metabolism in Health and Disease. *Nutrients* 12, (2019). 10.3390/nu12010094 PMC7019254 31905727

[29] Tsereteli, N. et al. Impact of insufficient sleep on dysregulated blood glucose control under standardised meal conditions. *Diabetologia*10.1007/s00125-021-05608-y (2021). 10.1007/s00125-021-05608-y PMC8741723 34845532

[30] Burdakov D (2007). K+ channels stimulated by glucose: a new energy-sensing pathway. Pflug. Arch..

[31] Wyatt P (2021). Postprandial glycaemic dips predict appetite and energy intake in healthy individuals. Nat. Metab..

[32] Holt SH, Delargy HJ, Lawton CL, Blundell JE (1999). The effects of high-carbohydrate vs high-fat breakfasts on feelings of fullness and alertness, and subsequent food intake. Int. J. Food Sci. Nutr..

[33] Anderson C, Horne JA (2006). A high sugar content, low caffeine drink does not alleviate sleepiness but may worsen it. Hum. Psychopharmacol..

[34] Edefonti V (2014). The effect of breakfast composition and energy contribution on cognitive and academic performance: a systematic review. Am. J. Clin. Nutr..

[35] Afaghi A, O’Connor H, Chow CM (2007). High-glycemic-index carbohydrate meals shorten sleep onset. Am. J. Clin. Nutr..

[36] Kim SW, Lee BI (2009). Metabolic state, neurohormones, and vagal stimulation, not increased serotonin, orchestrate postprandial drowsiness. Biosci. Hypotheses.

[37] Luneke, A. C. et al. Energy drink expectancies among college students. *J Am. Coll. Health* 1–9 10.1080/07448481.2020.1790569 (2020). 10.1080/07448481.2020.1790569 32673177

[38] Kreibig SD (2010). Autonomic nervous system activity in emotion: a review. Biol. Psychol..

[39] Young SN (2007). How to increase serotonin in the human brain without drugs. J. Psychiatry Neurosci..

[40] Oken BS, Salinsky MC, Elsas SM (2006). Vigilance, alertness, or sustained attention: physiological basis and measurement. Clin. Neurophysiol..

[41] Jackowska M, Ronaldson A, Brown J, Steptoe A (2016). Biological and psychological correlates of self-reported and objective sleep measures. J. Psychosom. Res..

[42] Ben Simon, E., Vallat, R., Barnes, C. M. & Walker, M. P. Sleep loss and the socio-emotional brain. *Trends Cogn. Sci*. **24**, 435–450 (2020). 10.1016/j.tics.2020.02.003 32299657

[43] Gobin CM, Banks JB, Fins AI, Tartar JL (2015). Poor sleep quality is associated with a negative cognitive bias and decreased sustained attention. J. Sleep. Res..

[44] Hoevenaar-Blom MP, Spijkerman AMW, Kromhout D, van den Berg JF, Verschuren WMM (2011). Sleep duration and sleep quality in relation to 12-year cardiovascular disease incidence: the MORGEN study. Sleep.

[45] Martin JL (2011). Poor self-reported sleep quality predicts mortality within one year of inpatient post-acute rehabilitation among older adults. Sleep.

[46] Drapeau C, Carrier J (2004). Fluctuation of waking electroencephalogram and subjective alertness during a 25-hour sleep-deprivation episode in young and middle-aged subjects. Sleep.

[47] Leproult R (2003). Individual differences in subjective and objective alertness during sleep deprivation are stable and unrelated. Am. J. Physiol. Regul. Integr. Comp. Physiol..

[48] Finelli LA, Baumann H, Borbély AA, Achermann P (2000). Dual electroencephalogram markers of human sleep homeostasis: correlation between theta activity in waking and slow-wave activity in sleep. Neuroscience.

[49] van Hees VT (2018). Estimating sleep parameters using an accelerometer without sleep diary. Sci. Rep..

[50] Petrowski K, Schmalbach B, Niedling M, Stalder T (2019). The effects of post-awakening light exposure on the cortisol awakening response in healthy male individuals. Psychoneuroendocrinology.

[51] Souman JL, Tinga AM, Te Pas SF, van Ee R, Vlaskamp BNS (2018). Acute alerting effects of light: A systematic literature review. Behav. Brain Res..

[52] Asnicar, F. et al. Microbiome connections with host metabolism and habitual diet from 1,098 deeply phenotyped individuals. *Nat. Med*. 10.1038/s41591-020-01183-8 (2021). 10.1038/s41591-020-01183-8 PMC8353542 33432175

[53] Buysse DJ, Reynolds CF, Monk TH, Berman SR, Kupfer DJ (1989). The Pittsburgh Sleep Quality Index: a new instrument for psychiatric practice and research. Psychiatry Res..

[54] Wittmann M, Dinich J, Merrow M, Roenneberg T (2006). Social jetlag: misalignment of biological and social time. Chronobiol. Int..

[55] Kolovou GD (2011). Assessment and clinical relevance of non-fasting and postprandial triglycerides: an expert panel statement. Curr. Vasc. Pharmacol..

[56] Wright KP, Hull JT, Czeisler CA (2002). Relationship between alertness, performance, and body temperature in humans. Am. J. Physiol. Regul. Integr. Comp. Physiol..

[57] Syed S, Morseth B, Hopstock LA, Horsch A (2020). Evaluating the performance of raw and epoch non-wear algorithms using multiple accelerometers and electrocardiogram recordings. Sci. Rep..

[58] van Hees, V. et al. *GGIR*. 10.5281/zenodo.3474227 (2019).

[59] Jones SE (2019). Genetic studies of accelerometer-based sleep measures yield new insights into human sleep behaviour. Nat. Commun..

[60] R Core Team. R: A language and environment for statistical computing. R Foundation for Statistical Computing, Vienna, Austria. https://www.R-project.org/ (2020).

[61] Bates D, Mächler M, Bolker B, Walker S (2015). Fitting linear mixed-effects models using lme4. J. Stat. Softw., Artic..

[62] Kuznetsova A (2017). lmerTest package: tests in linear mixed effects models. J. Stat. Softw..

[63] Lüdecke, D. sjPlot: Data visualization for statistics in social science. *R package version* 2, (2018).

[64] Lenth, R. V. emmeans: Estimated Marginal Means, aka Least-Squares Means. R package version 1.5.3. https://CRAN.R-project.org/package=emmeans (2020).

[65] Nakagawa S, Schielzeth H (2013). A general and simple method for obtaining R 2 from generalized linear mixed-effects models. Methods Ecol. Evol..

[66] Yu H, Jiang S, Land KC (2015). Multicollinearity in hierarchical linear models. Soc. Sci. Res..

[67] Yarkoni T, Westfall J (2017). Choosing prediction over explanation in psychology: lessons from machine learning. Perspect. Psychol. Sci..

[68] Lundberg SM (2020). From local explanations to global understanding with explainable AI for trees. Nat. Mach. Intell..

[69] Pedregosa F (2011). Scikit-learn: machine learning in python. J. Mach. Learn. Res..

[70] Jonsson H (2021). Differences between germline genomes of monozygotic twins. Nat. Genet..

[71] Purcell S (2002). Variance components models for gene–environment interaction in twin analysis. Twin Res. Hum. Genet..

[72] Scheike TH, Holst KK, Hjelmborg JB (2014). Estimating heritability for cause specific mortality based on twin studies. Lifetime Data Anal..

[73] Wang C, Roy-Gagnon M-H, Lefebvre J-F, Burkett KM, Dubois L (2019). Modeling gene-environment interactions in longitudinal family studies: a comparison of methods and their application to the association between the IGF pathway and childhood obesity. BMC Med. Genet..

