# Supplementary material for: How people wake up is associated with previous night’s sleep together with physical activity and food intake
Source: Nat Commun. 2022 Nov 19;13:7116. doi: 10.1038/s41467-022-34503-2 (PMC9675783; doi:10.1038/s41467-022-34503-2)
Supplement: Supplementary file 1 — Supplementary Information [file 41467_2022_34503_MOESM1_ESM.pdf]

How people wake up is associated with previous night's sleep together with physical activity and food intake

Raphael Vallat[1*], Sarah E. Berry[2], Neli Tsereteli[3], Joan Capdevila[4], Haya Al Khatib[2,4], Ana M. Valdes[5,6], Linda M. Delahanty[7], David A. Drew[8,9], Andrew T. Chan[8,9], Jonathan Wolf[4], Paul W. Franks[3,10,11], Tim D. Spector[10], Matthew P. Walker[1*]

Affiliations

[1] Center for Human Sleep Science, Department of Psychology, University of California, Berkeley, USA

[2] Department of Nutritional Sciences, King's College London, London, UK

[3] Department of Clinical Sciences, Lund University, Malmö, Sweden

[4] Zoe Ltd, London, UK

[5] School of Medicine, University of Nottingham, Nottingham, UK

[6] Nottingham NIHR Biomedical Research Centre, Nottingham, UK

[7] Diabetes Unit, Massachusetts General Hospital and Harvard Medical School, Boston, MA, USA

[8] Clinical & Translational Epidemiology Unit, Massachusetts General Hospital and Harvard Medical School, Boston, MA

[9] Division of Gastroenterology, Massachusetts General Hospital and Harvard Medical School, Boston, MA

[10] Department of Twin Research and Genetic Epidemiology, King's College London, London, UK

[11] Department of Nutrition, Harvard Chan School of Public Health, Boston, MA, USA

Corresponding authors

Raphael Vallat (raphaelvallat@berkeley.edu), Matthew P. Walker (mpwalker@berkeley.edu)

Department of Psychology, 2121 Berkeley Way, University of California, Berkeley, CA 94720-1650, USA. Phone: 510-642-5292 / Fax: 510-642-5293.

Supplementary Information

Supplementary Table 1. Predictors of day-to-day fluctuations in morning alertness.

Predictors	β	95% CI	p
Sleep (within-person, i.e. deviation from a person's average)			
Sleep efficiency	2.57	-4.62 – 9.76	0.483
Sleep duration	0.90	0.59 – 1.20	**<0.001**
Sleep offset	0.75	0.33 – 1.16	**<0.001**
Physical activity and circadian rhythm			
L5 value	-0.21	-0.36 – -0.07	**0.004**
L5 onset	-0.12	-0.34 – 0.10	0.289
M10 value (prior day)	0.02	0.00 – 0.03	**0.049**
M10 onset (prior day)	0.07	-0.09 – 0.22	0.401
Standardized breakfast meal, compared to reference "UK Average"			
High Carbohydrates	1.42	0.52 – 2.33	**0.002**
High Fat	-0.86	-1.93 – 0.22	0.119
High Fiber	-0.57	-1.67 – 0.53	0.311
High Protein	-1.36	-2.43 – -0.30	**0.012**
Metabolic Challenge	0.24	-1.12 – 1.60	0.730
OGTT	-6.97	-7.95 – -5.98	**<0.001**
Others meal-related predictors			
Post-breakfast glucose response	-2.89	-4.40 – -1.39	**<0.001**
Latency from sleep offset to breakfast	2.03	1.68 – 2.39	**<0.001**
Covariates			
Age	0.39	0.30 – 0.48	**<0.001**
DST	2.05	0.29 – 3.81	**0.022**
Weekend	-1.66	-2.34 – -0.99	**<0.001**
No. observations		6744	
Conditional R^2		0.626	

Sex, BMI, zygosity and sunrise time were included in the model but are not reported here for conciseness since none of them was a significant predictor of morning alertness. Sleep predictors were normalized using person-mean centering. The dependent variable of the model is morning alertness, which is calculated by averaging the alertness ratings that were made within the first

three hours after breakfast start. Family ID and participant ID were defined as nested random effects of the linear mixed model. P-values are based on two-tailed Wald tests (degrees of freedom = 6717) and are not adjusted for multiple comparisons. Significant p-values are reported in bold font. Source data are provided as a Source Data file. L5 = least active 5 hours of the day, M10 = most active 10 hours of the day, OGTT = Oral Glucose Tolerance Test.

Supplementary Table 2. Predictors of day-to-day fluctuations in morning alertness, using sleep onset instead of sleep offset.

Predictors	β	95% CI	p
Sleep (within-person, i.e. deviation from a person's average)			
Sleep efficiency	2.58	-4.61 – 9.77	0.482
Sleep duration	1.65	1.27 – 2.03	**<0.001**
Sleep onset	0.76	0.34 – 1.17	**<0.001**
No. observations		6744	
Conditional R^2		0.626	

Only the sleep predictors are reported here for conciseness. The other predictors are not reported because the significance or direction of the effects did not change compared to the main model (Supplementary Table S1). P-values are based on two-tailed Wald tests (degrees of freedom = 6717) and are not adjusted for multiple comparisons. Significant p-values are reported in bold font. Source data are provided as a Source Data file.

Supplementary Table 3. Predictors of day-to-day changes in morning alertness, excluding the Oral Glucose Tolerance Test from the standardized breakfast meals.

Predictors	β	95% CI	p
Sleep (within-person, i.e. deviation from a person's average)			
Sleep efficiency	3.95	-3.92 – 11.83	0.325
Sleep duration	0.89	0.56 – 1.22	**<0.001**
Sleep offset	0.45	0.00 – 0.91	**0.049**
Physical activity and circadian rhythm			
L5 value	-0.22	-0.37 – -0.06	**0.006**
L5 onset	-0.09	-0.33 – 0.15	0.446
M10 value (prior day)	0.02	-0.00 – 0.04	0.067
M10 onset (prior day)	-0.06	-0.23 – 0.11	0.478
Standardized breakfast meal, compared to reference "UK Average"			
High Carbohydrates	1.49	0.60 – 2.38	**0.001**
High Fat	-0.71	-1.78 – 0.35	0.188
High Fiber	-0.52	-1.59 – 0.55	0.340
High Protein	-1.30	-2.36 – -0.25	**0.015**
Metabolic Challenge	0.13	-1.20 – 1.46	0.849
Others meal-related predictors			
Post-breakfast glucose response	-2.59	-4.32 – -0.87	**0.003**
Latency from sleep offset to breakfast	1.66	1.25 – 2.07	**<0.001**
Covariates			
Age	0.38	0.29 – 0.47	**<0.001**
DST	1.77	-0.02 – 3.55	**0.052**
Weekend	-1.88	-2.61 – -1.14	**<0.001**
No. observations	5444		
Conditional R^2	0.625		

Sex, BMI, zygosity and sunrise time were included in the model but are not reported here for conciseness since none of them was a significant predictor of morning alertness. Sleep predictors were normalized using person-mean centering. The dependent variable of the model is morning alertness, which is calculated by averaging the alertness ratings that were made within the first three hours after breakfast start. Family ID and participant ID were defined as nested random

effects of the linear mixed model. P-values are based on two-tailed Wald tests (degrees of freedom = 5418) and are not adjusted for multiple comparisons. Significant p-values are reported in bold font. Source data are provided as a Source Data file. L5 = least active 5 hours of the day, M10 = most active 10 hours of the day.

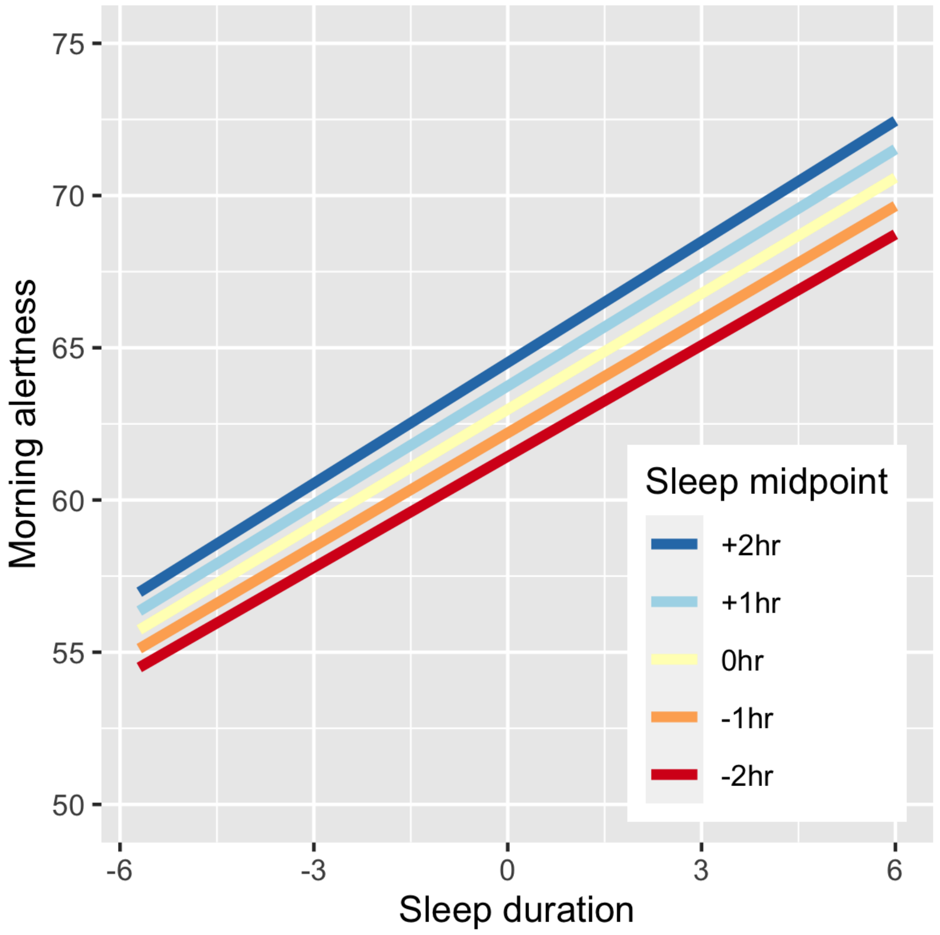

Supplementary Figure 1. The interaction of sleep duration and sleep timing is not significantly associated with next-morning alertness. Timing of sleep was measured using the sleep midpoint, i.e. the midpoint between the sleep onset and sleep offset. The significance of the interaction term and main effects of sleep duration and sleep timing was evaluated using a multilevel model (two-tailed Wald test with 6716 degrees of freedom). All sleep predictors were normalized using a person-mean centering. The main effects of sleep duration and sleep midpoint were both significant ($ps \leq 0.001$), however the interaction term was not significant ($p=0.792$). The interaction plot shows the regression line of sleep duration on morning alertness at different values of the moderator variable sleep midpoint, ranging from -2 hours to +2 hours in increments of 1 hour. All regression lines were significant ($p<0.001$), indicating that the positive impact of sleep duration on next-morning alertness was not moderated by the timing of sleep. Source data are provided as a Source Data file.

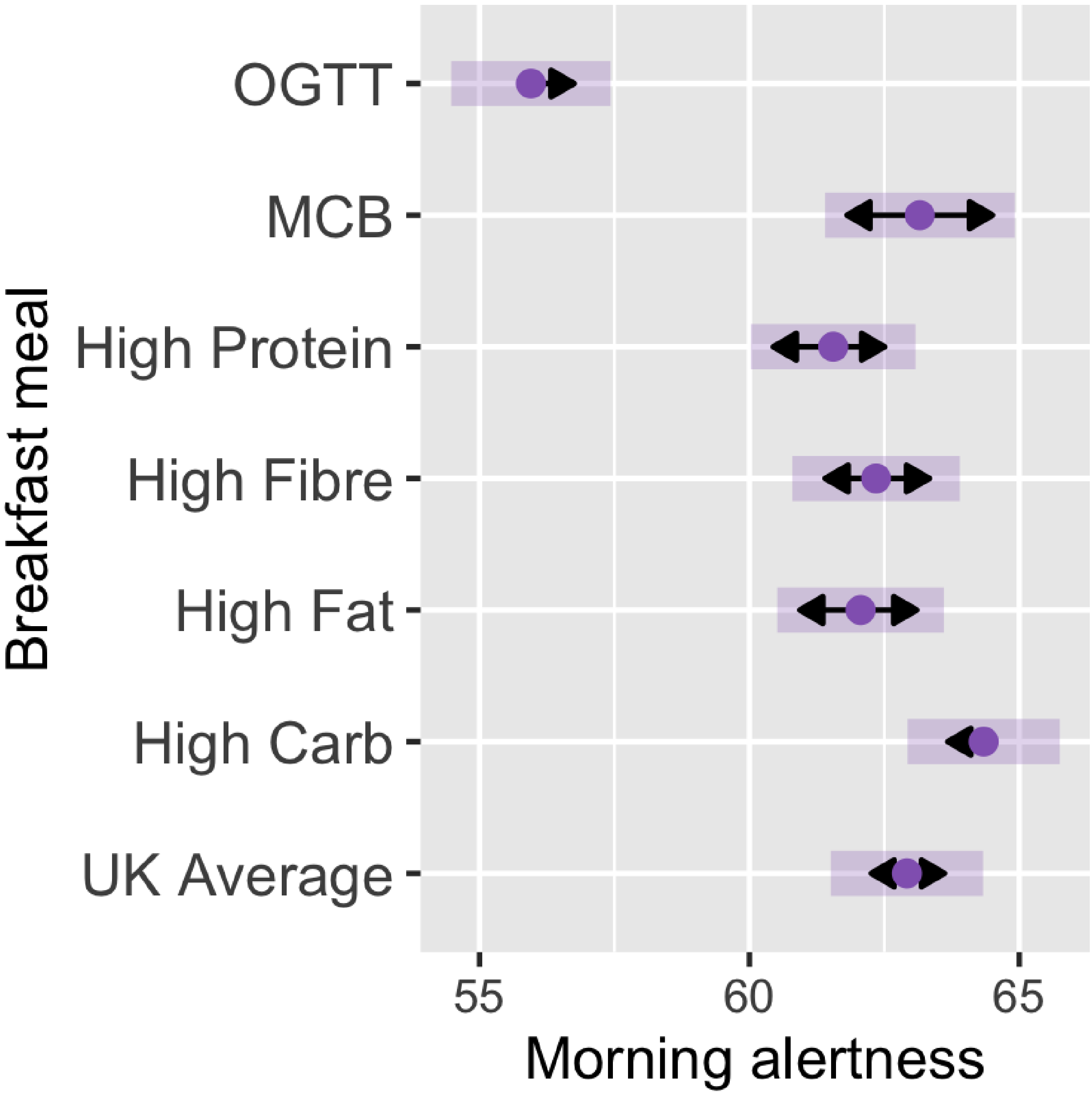

Supplementary Figure 2. Standardised breakfast meal and subsequent morning alertness. Purple dots and translucent rectangles show the estimated marginal means and 95% confidence intervals from the linear mixed effect model (Table S1). Black arrows represent significance, i.e. the degree to which two arrows overlap denotes the significance of the comparison of the two estimates. Two-tailed pairwise comparisons of the estimated marginal means were corrected for multiple comparisons using Tukey's method. Nutritional composition of each meal can be found in Table 1. Source data are provided as a Source Data file. OGTT = oral glucose tolerance test, MCB = Metabolic Challenge Breakfast.

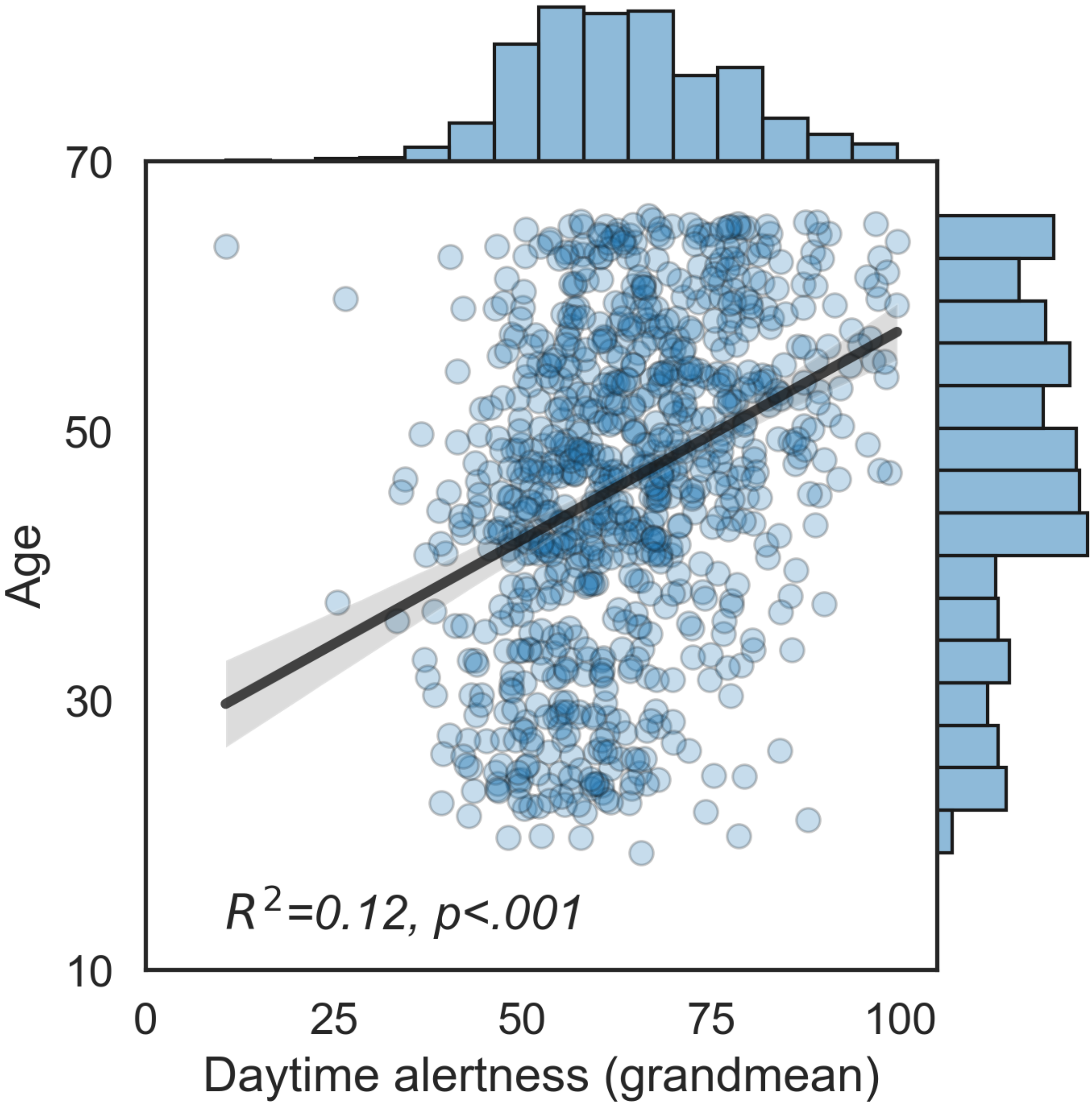

Supplementary Figure 3. Correlation between age and trait daytime alertness. Source data are provided as a Source Data file.

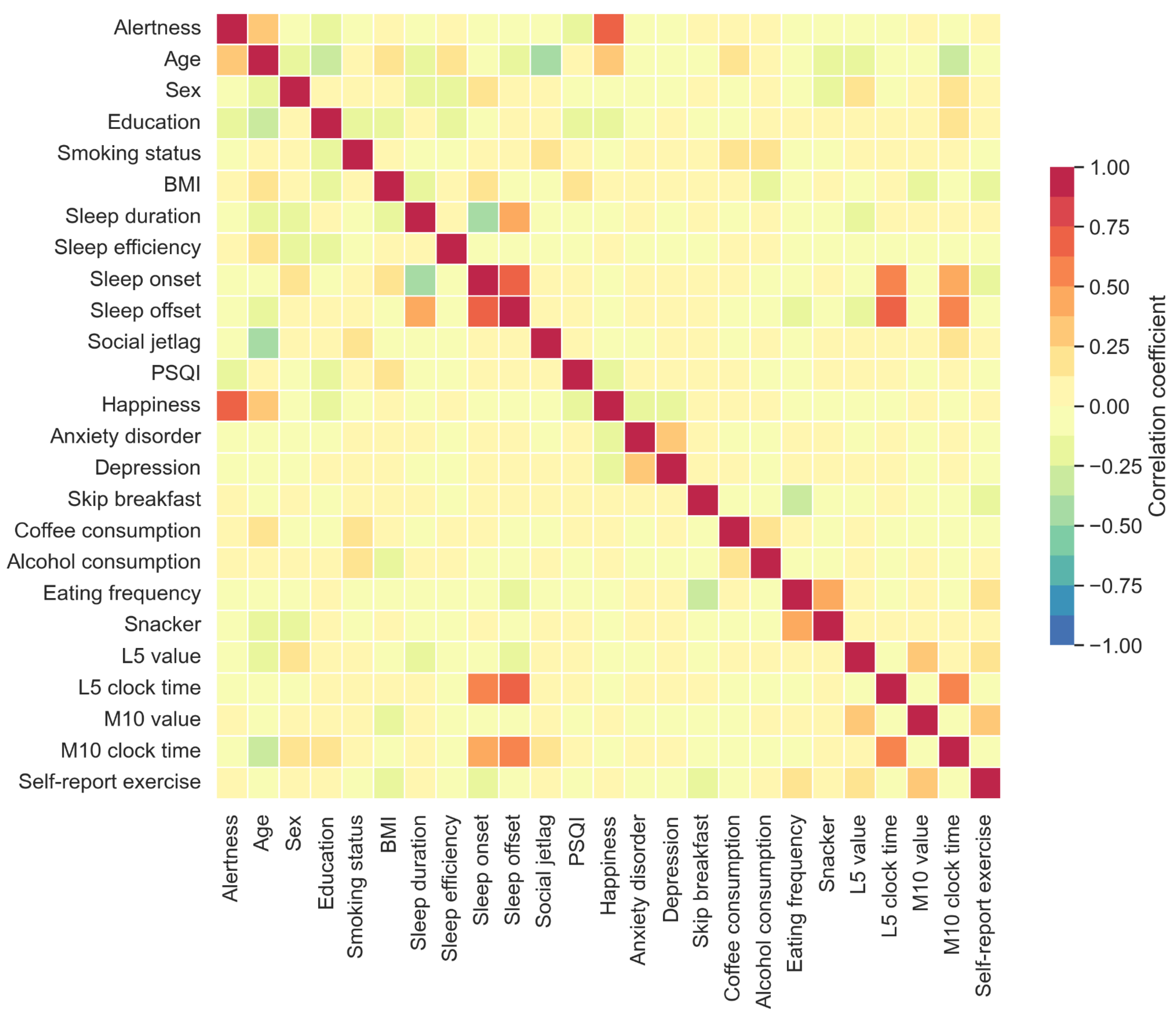

Supplementary Figure 4. Correlation matrix between alertness and trait predictors. Source data are provided as a Source Data file.

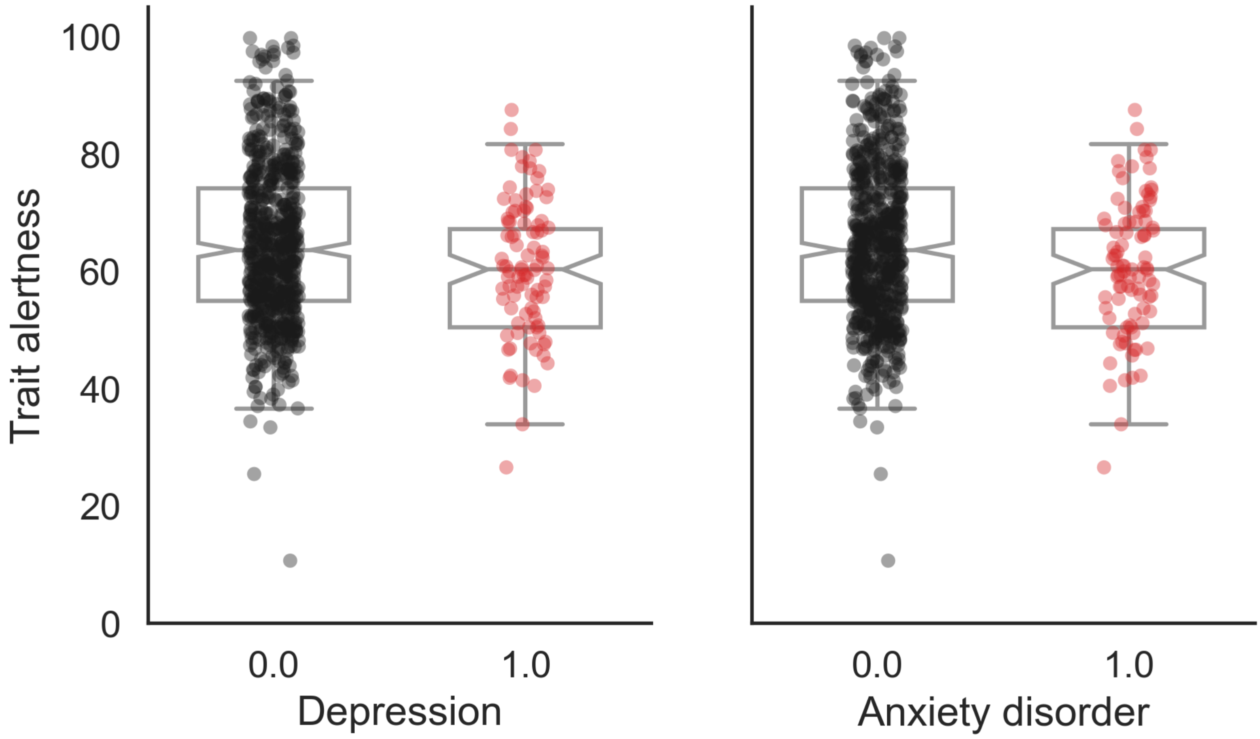

Supplementary Figure 5. Trait alertness is lower in participants with ongoing or former diagnosis of mood disorder. Sample sizes are: anxiety disorder (no [0] = 630, yes [1] = 113), depression (no [0] = 640, yes [1] = 93). Box plots show centre line as median, box limits as upper and lower quartiles. The notches represent confidence intervals around the median. The whiskers extend from the box limits by 1x the interquartile range. Source data are provided as a Source Data file.
