# Supplementary material for: How people wake up is associated with previous night’s sleep together with physical activity and food intake
Source: Nat Commun. 2022 Nov 19;13:7116. doi: 10.1038/s41467-022-34503-2 (PMC9675783; doi:10.1038/s41467-022-34503-2)
Supplement: Supplementary file 2 — Reporting Summary [file 41467_2022_34503_MOESM2_ESM.pdf]

nature portfolio

Corresponding author(s): Matthew P. Walker

Last updated by author(s): Sep 28, 2022

Reporting Summary

Nature Portfolio wishes to improve the reproducibility of the work that we publish. This form provides structure for consistency and transparency in reporting. For further information on Nature Portfolio policies, see our Editorial Policies and the Editorial Policy Checklist.

Statistics

For all statistical analyses, confirm that the following items are present in the figure legend, table legend, main text, or Methods section.

n/a	Confirmed	
☐	☒	The exact sample size (*n*) for each experimental group/condition, given as a discrete number and unit of measurement
☐	☒	A statement on whether measurements were taken from distinct samples or whether the same sample was measured repeatedly
☐	☒	The statistical test(s) used AND whether they are one- or two-sided<br>*Only common tests should be described solely by name; describe more complex techniques in the Methods section.*
☐	☒	A description of all covariates tested
☐	☒	A description of any assumptions or corrections, such as tests of normality and adjustment for multiple comparisons
☐	☒	A full description of the statistical parameters including central tendency (e.g. means) or other basic estimates (e.g. regression coefficient) AND variation (e.g. standard deviation) or associated estimates of uncertainty (e.g. confidence intervals)
☐	☒	For null hypothesis testing, the test statistic (e.g. *F*, *t*, *r*) with confidence intervals, effect sizes, degrees of freedom and *P* value noted<br>*Give P values as exact values whenever suitable.*
☒	☐	For Bayesian analysis, information on the choice of priors and Markov chain Monte Carlo settings
☐	☒	For hierarchical and complex designs, identification of the appropriate level for tests and full reporting of outcomes
☐	☒	Estimates of effect sizes (e.g. Cohen's *d*, Pearson's *r*), indicating how they were calculated

Our web collection on statistics for biologists contains articles on many of the points above.

Software and code

Policy information about availability of computer code

Data collection	No software was used for data collection
Data analysis	Raw accelerometer data was analyzed using the GGIR R package v1.10-7. All multilevel analyses were performed in R v4.0.2 using the lme4 v1.1-26, lmerTest 3.1-3 and sjPlot v2.8.7 packages. Heritability analyses were conducted using the mets v1.2.8.1 R package. Machine-learning analyses were conducted in Python 3.8 using the following packages: shap v0.40.0 , scikit-learn v1.0.2, lightgbm v3.31, and pingouin v0.5.1.

For manuscripts utilizing custom algorithms or software that are central to the research but not yet described in published literature, software must be made available to editors and reviewers. We strongly encourage code deposition in a community repository (e.g. GitHub). See the Nature Portfolio guidelines for submitting code & software for further information.

Data

Policy information about availability of data

All manuscripts must include a data availability statement. This statement should provide the following information, where applicable:

- Accession codes, unique identifiers, or web links for publicly available datasets
- A description of any restrictions on data availability
- For clinical datasets or third party data, please ensure that the statement adheres to our policy

The data of the at-home phase of the PREDICT1 trial, which supports the findings of this study, are held by Zoe Ltd. These data were used under licence for the current study and are therefore not publicly available. Data are however available from the authors upon reasonable request and with permission of Zoe Ltd.
The data of the baseline in-clinic visit of the PREDICT1 trial are held by the department of Twin Research at King's College London. The data can be released to bona fide researchers using normal procedures overseen by the Wellcome Trust and its guidelines as part of our core funding. We receive around 100 requests per year for our datasets and have a meeting three times a month with independent members to assess proposals. The application can be found at: https://twinsuk.ac.uk/

resources-for-researchers/access-our-data/. Data must be anonymized and conform to General Data Protection Regulation standards.
Source data are provided with this paper.

Field-specific reporting

Please select the one below that is the best fit for your research. If you are not sure, read the appropriate sections before making your selection.

☐ Life sciences ☒ Behavioural & social sciences ☐ Ecological, evolutionary & environmental sciences

For a reference copy of the document with all sections, see nature.com/documents/nr-reporting-summary-flat.pdf

Behavioural & social sciences study design

All studies must disclose on these points even when the disclosure is negative.

Study description

PREDICT 1 is a single-arm, single-blind longitudinal intervention study. The PREDICT 1 protocol is registered with the US National Institutes of Health trial registry (NCT03479866) and can be found at: https://clinicaltrials.gov/ct2/show/NCT03479866

Research sample

Study participants were healthy individuals aged 18–65 years, who were able to provide written informed consent. Exclusion criteria included ongoing inflammatory disease; cancer in the last three years (excluding skin cancer); long-term gastrointestinal disorders including irritable bowel disease or Celiac disease (gluten allergy), but not including irritable bowel syndrome; taking immunosuppressants or antibiotics as daily medication within the last three months; capillary glucose level of >12 mmol l–1 (or 216 mg dl–1), or type 1 diabetes mellitus, or taking medication for type 2 diabetes mellitus; currently experiencing acute clinically diagnosed depression; heart attack (myocardial infarction) or stroke in the last 6 months; pregnancy; and vegan or experiencing an eating disorder or unwilling to consume foods that are part of the study. Diagnosis or symptoms of any sleep disorders, circadian rhythm disorders, or neurocognitive disorders were not exclusionary. Similarly, the use of medication to impact sleep, circadian or brain function was not exclusionary.

A total of 970 generally healthy adults from the United Kingdom (including non-twins, monozygotic [MZ] twins and dizygotic [DZ] twins) as well as 95 healthy adults from the United States (all non-twins) were enrolled and completed baseline clinic measurements, as well as a two-week at-home phase.

Sampling strategy

1050 individuals were targeted for recruitment at baseline, which assumed a 5% loss-to-follow-up to correspond to 1000 evaluable individuals at the end of the study. The study was designed to be able to predict lipid and glucose postprandial responses based on individual characteristics, correlations of a magnitude of r=0.13 (R2=0.017) will be detected as statistically significant with p<0.005 and 80% power. Effects of r=0.165 (R2=0.027, i.e. explaining 2.7% of interindividual variation) are necessary to achieve 80% power with p<0.00001, i.e. accounting for 5000 independent hypothesis tests.

Data collection

Stool collection and questionnaires were completed at home and subsequently returned to study staff at the baseline visit. Those who could not collect a stool sample prior to their baseline visit completed the collection as soon as possible during the home-phase. Participants arrived at 8:30am for their visit and their written consent for participation was taken, after which they were cannulated in the forearm (antecubital vein) to collect a fasted blood sample before being fitted with wearable devices (continuous glucose monitor (CGM; Freestyle Libre Pro, Abbott, Abbott Park, IL, US) and wrist-based triaxial accelerometer (AX3, Axivity, Newcastle, UK)). Heart rate and blood pressure were measured using an automated blood pressure monitor while fasted (in triplicate, with mean of second and third measurements recorded). In a subgroup (n=456), ambulatory blood pressure was also monitored throughout the baseline visit (TM-2430, A&D Instruments Limited, Abingdon, UK). Participant weight, height, hip and waist circumference were measured using standard clinical techniques. Participants underwent a dual-energy, x-ray absorptiometry (DEXA) scan using a QDR Horizon W. Hologic Bone Densitometer (Vertec Scientific Ltd., Reading, UK). Fasting blood glucose level was checked using HemoCue Glucose 201 + System (Radiometer, Crawley, UK) and Stat Strip (Nova Biomedical, Waltham, MA, US) in the UK and US, respectively.

Upon completing their baseline visit, participants received all the components necessary to complete the home-phase as a take-home pack. The take-home pack contained: 1) Standardised meals for the home-phase; 2) DBS equipment and return envelopes (collection equipment plus 4x aluminium bags and one master pre-paid return envelope for returning these bags); 3) Stool sample collection kit; 4) Pre-paid return kit (box or envelope with 2 separate packs for stool and equipment) with postage-paid packs to return the activity monitor, the CGM and the stool sample.

During the study home-phase, participants consumed multiple standardised test meals over a 9-11 day period, differing in macronutrient composition (carbohydrate, fat, protein and fiber), while wearing the CGM and activity monitor. Participants recorded all of their dietary intake, satiety and exercise on the Zoe study app as outlined below throughout the study. DBS tests were completed on Days 2, 3, 4, and 5 before and after test meals. Participants also provided a stool sample upon completion of the study on Day 14. Following completion of the home-phase, participants returned all study samples and devices to study staff via standard mail.

Upon completing their clinical visit, participants received a pack of standardized test meals to take home for consumption, and were instructed on how to store and consume the meals. The study had a total of 3 test meal protocol groups. Standard test meals were consumed at breakfast during the first 9-11 days (Days 2-12) of the home period, and additionally for lunch on Days 2 and 3.

Participants received clear instructions (verbal and written) on how and when the meals were to be consumed and recorded in the Zoe study app, as described below. Standardized meals were provided frozen and participants were therefore instructed to store these in the freezer upon arriving at home. Each evening, participants were instructed to transfer the meals for the following day from the freezer to the refrigerator, so they were defrosted in time for consumption. Participants were instructed to fast for a minimum of 8 hours prior to consuming a test breakfast meal and to limit exercise and drink only still, not sparkling, water in

moderation during fasting periods (including during the overnight fast).

In addition to water, participants were allowed to have one tea or coffee with their muffin-based set breakfasts. Tea or coffee could contain up to 40 mL 0.1% (skimmed) fat cow's milk, and no sugar or other sweeteners were not allowed. If participants consumed coffee or tea with their breakfasts, they were instructed to consume this drink consistently, in the same strength and amount, alongside all muffin-based test meals throughout the study. Participants could choose to consume a single tea or coffee alongside their lunch meal, subject to the same restrictions as the breakfast drink, and were asked to keep this drink consistent between test lunches on the study. They were instructed to consume all muffin-based meals within 10 minutes and the OGTT meal within 5 minutes, and to notify study staff if this was not achieved. After consuming their breakfast, participants were instructed to fast for 3 or 4 hours after meal consumption (depending on test meal protocol; protocol Group 1 had a fasting period of 3 hours for Meal 5, and 4 hours for all other meals; in Groups 2 and 3 had a fasting period of 3 hours for all breakfast meals, excluding combinations of breakfast and lunch, where fasting periods were 4 and 2 hours, respectively). They were advised to limit exercise and drink only plain, still water and when possible and safe, to avoid taking any over-the-counter medication during fasting periods.

Participants were instructed to not consume any food or drink other than water alongside the OGTT (Meal 5), to refrain from movement or activity as much as possible for the first hour following the breakfast, and avoid physical activity during the 3-hour fasting period that followed it. Participants were allowed to consume other foods as they normally would once they had completed the fasting period on the study day. Test meals and any dietary intake consumed within fasting periods, including accompanying drinks, were recorded in the Zoe study app by participants with the exact time at consumption and ingredient quantities so that compliance could be monitored by study staff. Only test meals that were completed according to instructions were included in analysis.

Test meals were prepared and packaged in the Dietetics Kitchen (Department of Nutritional Sciences, King's College London, London, UK) using standard ingredients; plain flour, sugar, baking powder, vanilla essence, milk, egg, salt, high-oleic sunflower oil, whey protein powder, chocolate milkshake powder (Nesquik, Nestle, Gatwick, UK), and commercially available fiber bars (Chocolate Fudge Brownie, Fibre One, General Mills, MN, US; Goodness Bar Apple & Walnut, The Food Doctor, Hessle, UK). All test meal muffins were stored frozen until delivery to participants, who were instructed to store them frozen at home. Powder sachets and fiber bars were stored at room temperature until consumption, while long-life milk was stored at room temperature until delivery to the participant and then refrigerated in their home-phase. Test meals baked for use by the US cohort were shipped frozen, under temperature controlled-conditions, to the US to limit variability of the intervention. Test meal drinks were prepared by the participant at home by mixing pre-portioned powder sachets with long-life milk provided (Meal 1: Metabolic Challenge Meal, 220 mL 0.1% fat milk; Meal 8: High Protein, 200 mL 1.6% fat milk). The OGTT consisted of a pre-portioned powdered glucose sachet which participants mixed with 300 mL water in the UK. In the US, participants were provided with pre-mixed OGTTs ready for consumption (Cat# 82028-512; VWR, US).

Participants were blinded to the nutrient composition of test meals. Test meals were labelled with a barcode and randomization code to allow study staff to identify the composition of the meal consumed, but were also labelled with text for the participant as "Day 1", "Day 2", etc. so that they consumed meals on the proper day according to their randomisation scheme. To log their consumption of the test meal, participants were asked to scan the barcode with the Zoe study app immediately before consumption.

The Zoe study app was developed to support the PREDICT 1 study by serving as an electronic notebook of study tasks, a tool for recording all dietary intake and a portal for communication with study staff. The app sent participants notifications and reminders to complete tasks at certain time-points, such as when their test lunch meals and DBS were due. The app also prompted participants to report their hunger and alertness levels on visual analogue scales truncated from Flint et al8, by displaying the questions "how hungry are you?" and "how alert are you?" above the scales, at 0 minutes (time of logging) and regular intervals thereafter following the logging of a breakfast, lunch or dinner meal. Participants were asked to log in the study app any exercise which would not be well captured by a wrist-affixed accelerometer, such as cycling or weight-lifting, where their wrists were stationary during the exercise. Participants logged their full dietary intake using the study app over the 14-day study period, including all standardized test meals and free-living foods, beverages (including water) and medications. Data logged into the Zoe study app was uploaded onto a digital dashboard in real time and reviewed and assessed for logging accuracy and study guideline compliance by study staff.

Interstitial glucose was measured every 15 minutes using Freestyle Libre Pro continuous glucose monitors (Abbott, Abbott Park, IL, US). Monitors were fitted by trained clinical practitioners on the upper, non-dominant arm at participants' baseline clinical visit and covered with Opsite Flexifix adhesive film (Smith & Nephew Medical Ltd, Hull, England) for improved durability. CGMs were worn for the entire study duration (14 days) and removed on Day 15, after which they were mailed back to study staff. Given the CGM device requires time to calibrate once fitted to a participant, CGM data collected 12 hours and onwards after activating the device was used for analysis. To understand the accuracy of the CGM device, a subgroup of participants (n=377) were fitted with two monitors on the same arm. The Coefficient of Variation (CV =11.75%) was small and the correlation (r = 0.97) was high (for glucoseiAUC0-2h).

Energy expenditure was measured using a triaxial accelerometer (AX3, Axivity, Newcastle Upon Tyne, UK) fitted by clinical practitioners at the baseline clinic visit on the non-dominant wrist and worn for the duration of the study (except during water-based activities, including showers and swimming), after which they were removed on Day 15 and mailed back to study staff. Accelerometers were programmed to measure acceleration at 50 Hz with a dynamic range of ±8 g (where g refers to local gravitational force equal to 9.8 m/s2).

Timing

The initial study commenced in June 2018 in the United Kingdom. In December 2018, an independent trial mirroring the UK protocol was launched in parallel at Massachusetts General Hospital to serve as a US-based validation cohort. The studies were completed in May 2019.

Data exclusions

Full inclusion and exclusion criteria of the PREDICT1 clinical trial are included in Table 1 of the online protocol (https://clinicaltrials.gov/ct2/show/NCT03479866). Specific to this study, for both the meals and alertness data, participants with less than 5 days of valid data were removed. Second, participants that traveled in a different timezone during the two weeks of the home-based study were also excluded. Furthermore, a set of thresholds was then applied to remove invalid nights or participants, consistent with typical practices. First, nights with a TST outside the range of 2 to 15 hours, or a SE below 20%, were excluded. Second, nights with more than 10% of accelerometer epochs classified as invalid were excluded. Third, nights with a sleep onset between 8 AM and 5 PM

or a sleep offset after 12 PM were excluded.

Non-participation	The consort diagrams of the PREDICT1 clinical trial can be found in Figure 2 of the online protocol: https://protocolexchange.researchsquare.com/article/pex-802/v1<br><br>In the UK, there were 1002 participants enrolled, of which 20 withdrew their participation and 15 were lost to follow-up, resulting in a final sample size of 967. In the US, there were 100 participants enrolled, of which 4 withdrew their participation and 1 were lost to follow-up, resulting in a final sample size of 95.
Randomization	Meal order in each group was randomised using Microsoft Access for each participant, using a 2-block randomisation and 1 non-randomised block as denoted in Table 4 of the online protocol (https://clinicaltrials.gov/ct2/show/NCT03479866). Participants were blinded to the nutrient composition of test meals.

Reporting for specific materials, systems and methods

We require information from authors about some types of materials, experimental systems and methods used in many studies. Here, indicate whether each material, system or method listed is relevant to your study. If you are not sure if a list item applies to your research, read the appropriate section before selecting a response.

Materials & experimental systems

n/a	Involved in the study	
☒	☐	Antibodies
☒	☐	Eukaryotic cell lines
☒	☐	Palaeontology and archaeology
☒	☐	Animals and other organisms
☐	☒	Human research participants
☐	☒	Clinical data
☒	☐	Dual use research of concern

Methods

n/a	Involved in the study	
☒	☐	ChIP-seq
☒	☐	Flow cytometry
☒	☐	MRI-based neuroimaging

Human research participants

Policy information about studies involving human research participants

Population characteristics	See above
Recruitment	In the UK, participants (target n = 1,000) were recruited from the TwinsUK cohort, an ongoing research cohort described elsewhere and the general population using online advertising. In the US, participants (target n = 100) were recruited through online advertising, research participant databases and Rally for Research (https://rally.partners.org/), an online recruiting portal for research trials. During an initial recruitment interaction with study staff, participants were screened minimally for eligibility criteria (e.g. phone compatibility for the logging app, pregnancy, food allergies to test meal components) and to answer any questions from participants. If potential subjects were interested in participating and remained eligible, they then underwent a detailed eligibility assessment performed by a study coordinator over the phone. Participants were provided with an informational booklet as a "Participant Information Sheet" that fully described the study including both the clinical and at-home phase of the study to assist in the informed consent process.<br><br>As with most volunteer studies, there may have been a bias to enroll more health-conscious individuals than the general population. Therefore, participants may not be representative of a pure random sample of the US and UK population. However, since the study focused on within-individual analyses and adjusted for numerous covariates associated with health, this is unlikely to invalidate our scientific conclusions.
Ethics oversight	Ethical approval for the study was obtained in the UK from the Research Ethics Committee and Integrated Research Application System (IRAS 236407) and in the US from the Institutional Review Board (Partners Healthcare IRB 2018P002078). The trial was registered on ClinicalTrials.gov(registration number: NCT03479866) and was run in accordance with the Declaration of Helsinki and Good Clinical Practice. Study procedures were only carried out after having received written informed consent from each participant.

Note that full information on the approval of the study protocol must also be provided in the manuscript.

Clinical data

Policy information about clinical studies

All manuscripts should comply with the ICMJE guidelines for publication of clinical research and a completed CONSORT checklist must be included with all submissions.

Clinical trial registration	NCT03479866
Study protocol	https://clinicaltrials.gov/ct2/show/NCT03479866
Data collection	The full description of the experimental procedure can be found at: https://clinicaltrials.gov/ct2/show/NCT03479866

Data collection

The PREDICT 1 study was a two-country study conducted between June 2018 and May 2019, with the primary cohort based at St. Thomas' Hospital in London, UK and a validation cohort (that underwent the same profiling as in the UK) assessed at Massachusetts General Hospital (MGH) in Boston, Massachusetts.

The initial study commenced in June 2018 in the United Kingdom. In December 2018, an independent trial mirroring the UK protocol was launched in parallel at Massachusetts General Hospital to serve as a US-based validation cohort. The studies were completed in May 2019.

Outcomes

This trial aimed to examine the factors that predict individual postprandial responses in context of the individual and meals consumed. For primary outcome reporting, the study focused on the following postprandial metabolic response between 0 and 6 hours for blood triglycerides, glucose, and insulin concentrations to the sequential mixed-nutrient dietary challenges during the clinical visit on Day 1. A summary of all endpoints that were collected and considered as a priori outcomes of the study are presented in Table 5 of the online protocol.
